# Gatekeeping Activity of Collinear Ketosynthase Domains Limits Product Diversity for Engineered Type I Polyketide Synthases

**DOI:** 10.1021/acs.biochem.4c00249

**Published:** 2024-08-26

**Authors:** Dongqi Yi, Mujeeb A. Wakeel, Vinayak Agarwal

**Affiliations:** † School of Chemistry and Biochemistry, Georgia Institute of Technology, Atlanta, Georgia 30332, United States; ‡ School of Biological Sciences, Georgia Institute of Technology, Atlanta, Georgia 30332, United States

## Abstract

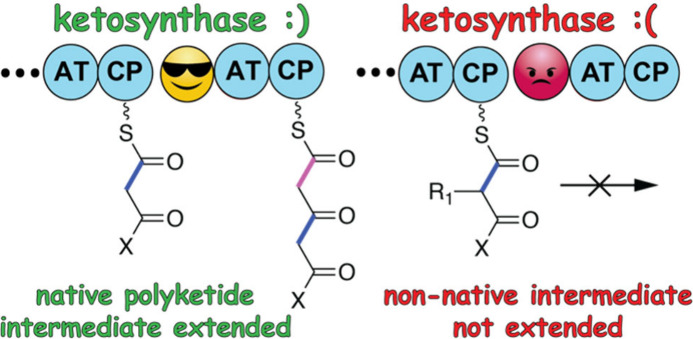

Engineered type I polyketide synthases (type I PKSs) can enable access to diverse polyketide pharmacophores and generate non-natural natural products. However, the promise of type I PKS engineering remains modestly realized at best. Here, we report that ketosynthase (KS) domains, the key carbon–carbon bond-forming catalysts, control which intermediates are allowed to progress along the PKS assembly lines and which intermediates are excluded. Using bimodular PKSs, we demonstrate that KSs can be exquisitely selective for the upstream polyketide substrate while retaining promiscuity for the extender unit that they incorporate. It is then the downstream KS that acts as a gatekeeper to ensure the fidelity of the extender unit incorporation by the upstream KS. We also demonstrate that these findings are not universally applicable; substrate-tolerant KSs do allow engineered polyketide intermediates to be extended. Our results demonstrate the utility for evaluating the KS-induced bottlenecks to gauge the feasibility of engineering PKS assembly lines.

Type I polyketide synthases (type I PKSs) catalyze the production of structurally intriguing and pharmaceutically valuable polyketide natural products. The utility of their products has generated interest in engineering type I PKSs to diversify polyketide pharmacophores. However, type I PKS engineering has been encumbered by our incomplete understanding of the molecular determinants for intermediate transitions through the PKS assembly lines.^1^

Type I PKSs operate in an assembly line wherein dicarboxylic acid building blocks are joined sequentially to a growing polyketide chain (Figure 1).^2^ Polyketide intermediates are thioesterified to the phosphopantetheinyl arm on carrier protein (CP) domains; the acyltransferase (AT) domains select the (substituted)malonyl extender units; the KS domains perform the carbon–carbon bond forming reaction by the decarboxylative Claisen condensation of the malonyl extender unit with the upstream polyketide intermediate; and combinations of the ketoreductase (KR), dehydratase (DH), and enoyl reductase (ER) domains tailor the β-carbonyl of the growing polyketide chain (Figure 1). Finally, a thioesterase (TE) domain offloads the polyketide natural product.^3^

**Figure 1:**
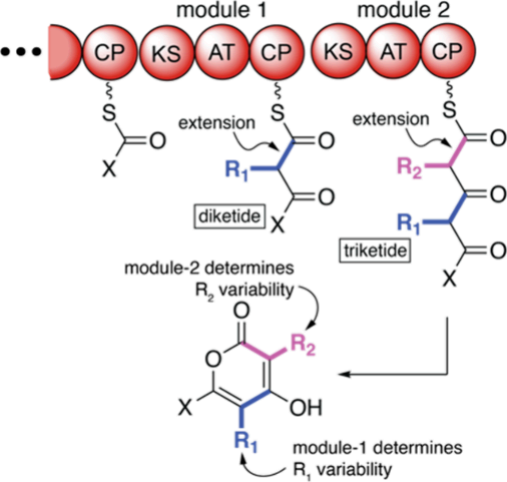
Modular architecture illustrated for a type I PKS furnishing an α-pyrone product.

From an engineering point-of-view, the AT domains have attracted intense interest as they select the extender units that are incorporated into the polyketide chain. By reprogramming the AT domains, incorporation of novel extender units into polyketide scaffolds was enabled, such as the production of fluorinated polyketides.^4−6^ Another engineering route has been module shuffling, in which heterologous modules that incorporate varied extender units are used to create designer PKS assembly lines.^7−9^ However, the productivity of engineered PKSs is often compromised. One of the primary limiting factors here has been the nonphysiological intermolecular protein–protein interactions between the native and the heterologous PKS modules/domains.

In addition to the AT domains, recently, a growing appreciation for the role of the KS domains in PKS engineering has developed. Three recent studies have precipitated this advance. First, by mutagenizing a KS active site, Leadlay expanded the diversity of diketide substrates that were elongated to triketide products by a single module PKS.^10^ Using shuffled modules of the 6-deoxyerythronolide B synthase (DEBS), Grininger demonstrated that mutagenizing the KS domain active site also improved the output of engineered PKSs.^11^ These findings were followed by a bioinformatic mapping of KS active site residues by Keatinge-Clay which could facilitate mutagenic engineering of KS domains.^12^ In this study, using modules derived from PKSs that furnish pyoluteorin and calcimycin polyketide natural products (henceforth referred to as the Plt and Cal PKSs, respectively),^13,14^ we demonstrate the gatekeeping role of intermediary collinear KS domains such that non-native polyketide intermediates are not allowed to progress along the PKS assembly line. We show that even if the substrate scope of a *cis*-AT domain is expanded, the selectivity of the KS domain remains a crucial bottleneck for the success of PKS engineering efforts.

First, we designed four PKS assembly lines from the Plt and Cal PKSs (Figure S1). The Plt PKS has been reconstituted *in vitro*.^15^ We have previously described the production of the triketide α-pyrone **1** when the thioesterified carboxylic acid—4,5-dichloropyrrolyl-*S*,*N*-acetylcysteamine (4,5-dichloropyrrolyl-SNAC)—was provided to the first two Plt PKS modules with the interdomain linkers from DEBS facilitating intermodular protein–protein interactions (Figures 2A, S2–S3).^16^ The physiological substrate for the Plt module-1 AT domain is malonyl-CoA (Mal-CoA) that was produced by the ATP-dependent condensation of malonic acid with CoA-SH by the enzyme MatB (Figure S4).^17^ The Plt module-2 AT domain is atypical in architecture and may be nonfunctional (henceforth referred to as AT*). It is then conceivable that the Plt module-1 AT is able to charge both, the cognate Plt module-1 CP, and the downstream Plt module-2 CP akin to Hertweck’s finding of the iterative CP-loading activity of the aureothin producing Aur PKS module-3 AT.^18^

**Figure 2:**
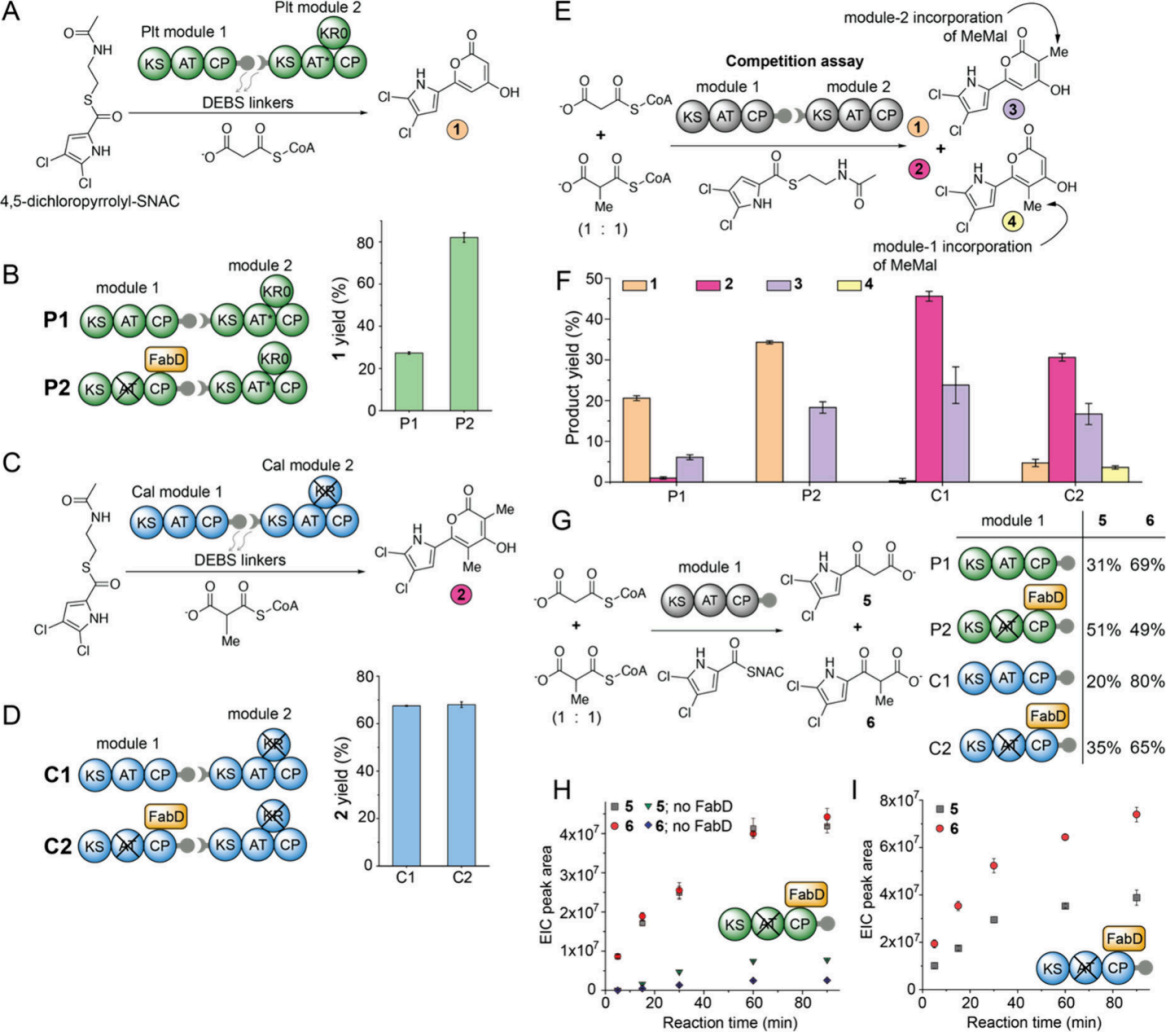
Design and evaluation of PKS assembly lines. (**A**) The Plt P1 PKS. The nonfunctional KR domain is labeled KR0. (**B**) P2 PKS in which the module-1 AT has been inactivated and FabD is added. Product yields are evaluated relative to the stoichiometry of the 4,5-dichloropyrrolyl-SNAC substrate added to the assay. (**C**) Cal C1 PKS. (**D**) The C1 and C2 PKSs. No difference in yield of **2** is observed between C1 and C2 PKSs. (**E**) Competition experiment between Mal and MeMal extender units leading to products **1**–**4**. (**F**) Yields of **1**–**4** produced in the competition assays. (**G**) Assay design and relative amounts of diketides **5** and **6** produced by the four unimodular PKSs. Time-dependent formation of **5** and **6** by (**H**) P2 and (**I**) C2 PKSs.

We refer to the PKS assembly line containing the wild type Plt module-1 and module-2 as “P1” (Figure 2B). Note that the production of **1** does not require the participation of a KR or a TE domain.^16^ Next, we inactivated Plt module-1 AT domain by active site mutagenesis and added the *trans*-acting *Escherichia coli* malonyl-CoA:CP transacylase FabD to the assay as a surrogate; this PKS is henceforth referenced to as “P2”. We have previously demonstrated that FabD can substitute for inactivated *cis*-acting AT domains and it is likely that FabD loads both, module-1 CP, and the module-2 CP in the P2 PKS.^16^

We next prepared PKS assembly lines from the Cal PKS. The thiotemplated 4,5-dichloropyrrolyl carboxylic acid is a competent substrate for the Cal module-1.^19^ The Cal PKS module-2 KR domain was inactivated by active site mutagenesis. The physiological substrate for the Cal module-1 and module-2 AT domains is methylmalonyl-CoA (MeMal-CoA), which was produced by using MatB (Figure S5). Using this PKS, which we term “C1”, we observed the production of the α-pyrone **2** (Figures 2C, S2, S6). Analogous to P2, the Cal module-1 AT was inactivated and replaced with FabD to yield C2 PKS (Figure S7). Mal- and MeMal-CoA are both competent substrates for FabD.^6^

Next, we performed a competition experiment in which Mal-CoA and MeMal-CoA were both provided together in an equimolar ratio to the above-mentioned PKSs (Figure 2E). In this competition assay, in addition to **1** and **2**, two other products, **3** (where only module-2 incorporates the MeMal extender unit) and **4** (where module-1 incorporates the MeMal extender unit) were produced (Figure S8). The relative product yields for the four PKSs were evaluated (Figures 2F, S9–S10). For PKSs P1 and P2, we observed the production of **1**, the physiological product, in the highest yield, followed by that of **3**. As before, P2 offered a higher yield of **1** and **3** as compared to that of the P1 PKS. The production of **2** and **4**, in which Plt module-1 would incorporate the MeMal extender unit, was not observed. The product profiles for the C1 and the C2 PKSs were different. Here, product **2** dominated, wherein both Cal modules would incorporate the MeMal extender units in line with their physiological activity,^14^ followed by product **3** in which module-1 of the C1 and C2 PKSs would incorporate the nonphysiological Mal extender unit.

The lack of formation of **2** and **4** by the Plt PKSs can be rationalized to occur via three possible mechanisms. First, the Plt module-1 *cis*-AT could be selective for Mal-CoA and exclude MeMal-CoA from being incorporated. However, if this was the specificity determining the reason, then we should have observed production of **2** and **4** by P2, which is not the case. Second, it is possible that the substrate selectivity of Plt module-1 KS would not allow for diketide formation when the module-1 CP is loaded with the non-native MeMal extender unit by either the module-1 AT or by FabD. In this scenario, the Plt module-1 KS would possess strict extender unit selectivity for the chain extension reaction. Third, it is plausible that the Plt module-2 KS is selective such that further extension of the MeMal-extended diketide is excluded by the module 2-KS. Note that the production of **3** by P1 and P2 PKSs demonstrates that the Plt module-2 KS is not selective for the module-2 extender unit.

To differentiate between the selectivity of the Plt module-1 KS versus that of the module-2 KS, we monitored diketide formation by module-1 *only* of the P1, P2, C1, and C2 PKSs (Figures 2G, S11, Table S1). The modules were challenged by equimolar ratios of Mal-CoA and MeMal-CoA in a competition experiment, and the formation of the diketide products **5** and **6** was monitored. Here, we discerned that the Plt module-1 KS could efficiently use the MeMal extender unit for diketide production. This assertion is borne out by the higher abundance of diketide **6** relative to **5** produced by the P1 module-1, and near equal abundances of **5** and **6** for the P2 module-1 (Figure 2G). Monitoring the time-dependent formation of **5** and **6** by P2 module-1 additionally shows no differences in the rate of the appearance of these two products (Figure 2H). Furthermore, we verified that recombinantly expressed and purified module-1 proteins did not suffer from endogenous contamination with *E. coli* FabD; the productivity of module-1 in the absence of exogenous FabD addition was reduced (Figure 2H).

These data imply that the lack of production of **2** and **4** by P1 and P2 PKSs could not be attributed to the selectivity of the Plt module-1 KS against the MeMal extender unit; it should instead be attributed to the diketide substrate selectivity of the Plt module-2 KS, in that the module-2 KS does not allow for a diketide intermediate that was extended upstream using a noncognate extender unit to progress along the assembly line. The Plt module-2 KS selectivity for the upstream polyketide intermediate is in consonance with the description of the substrate selectivity of the Plt module-1 KS for its own ketide substrate.^19^ Taken together, we posit that while the module-1 KS is selective for its ketide substrate, it is not selective for the extender unit; it is the module-2 KS that then gatekeeps for the correct extender unit being incorporated in Plt module-1. The module-1 AT is substrate promiscuous and exerts no control over the product profile by itself.

Unlike the Plt module-1 KS and AT domains, the Cal module-1 KS and AT domains do demonstrate a preference for their cognate MeMal extender unit as evidenced by the 4-fold higher abundance of **6** relative to **5** produced by the C1 module-1 (Figure 2G). When Cal module-1 AT was replaced with FabD, C2 module-1 still produced a higher abundance of **6**. Monitoring the time-dependent on **5** and **6** in a competition experiment established that the Cal module-1 KS did prefer performing the extension reaction using the cognate extender unit (MeMal leading to **6**) as opposed to the noncognate extender unit (Mal leading to **5**; Figure 2I). Within the biosynthetic milieu inside a bacterial cell, the incorporation of a MeMal extender unit is in competition with the primary metabolite Mal-CoA; it is thus conceivable that the Cal module-1 KS and AT domains work synergistically to generate specificity for the incorporation of MeMal into the diketide intermediate.

To demonstrate the relative effect of KS gatekeeping versus AT promiscuity on efforts to engineer diverse polyketide natural products, we expanded upon the extender unit competition experiments. As illustrated in Figure 3A, we refer to “Group A” pyrone products in which module-1 incorporates the cognate extender unit (Mal for P1 and P2; MeMal for C1 and C2). The “Group B” pyrones refer to products in which module-1 incorporates a non-native extender unit. An equimolar mixture of the cognate (R_1_, Figure 3A), and one each of the noncognate extender units (R_2_) were provided to set up the competition assay. In addition to Mal and MeMal, ethylmalonyl– (EtMal), and propargylmalonyl-CoA (PgMal) extender units were used (Figures S12–S15).

**Figure 3:**
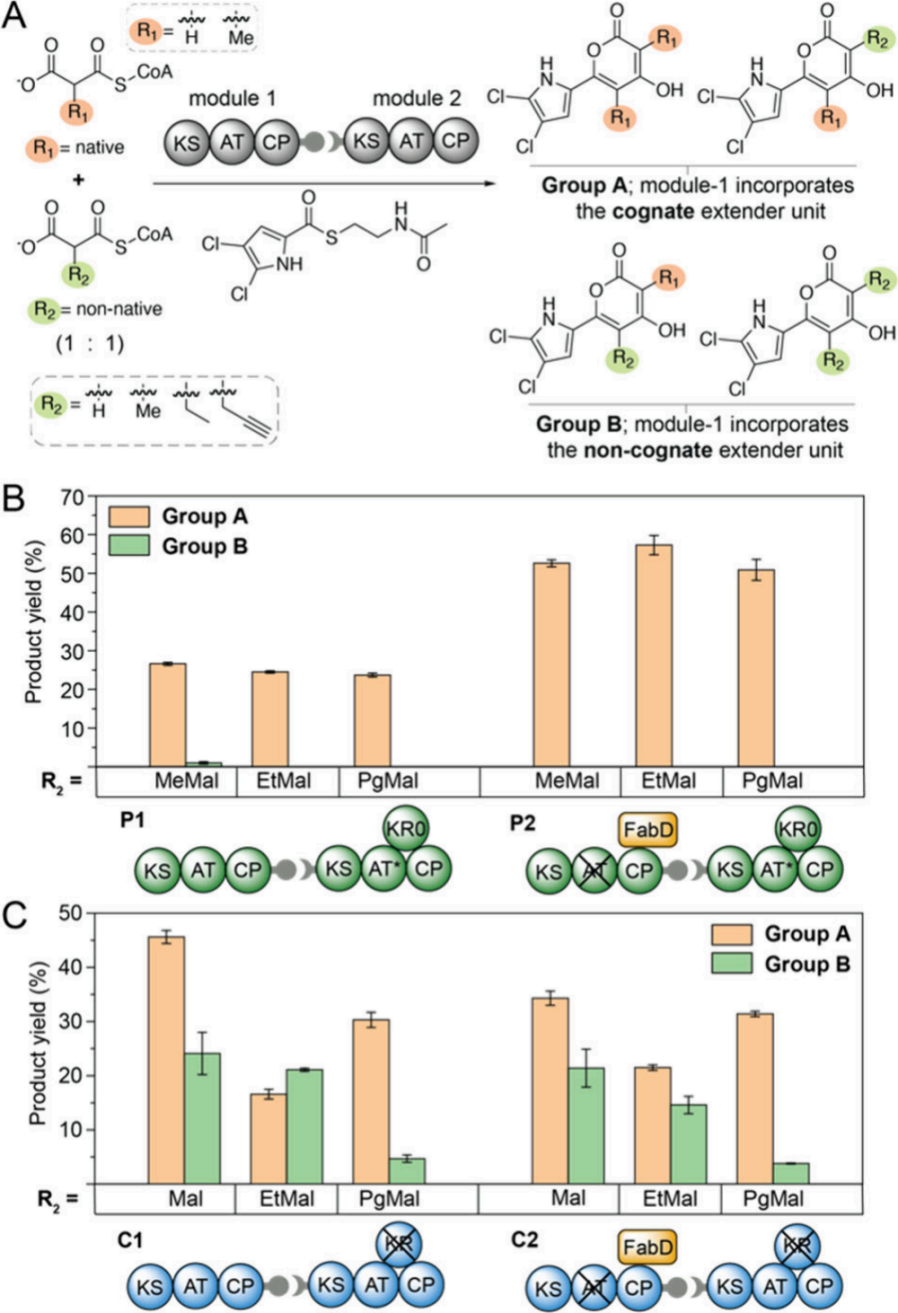
Expanded competition experiment. (**A**) Assay design in which four extender units are provided to the P1–P2 and C1–C2 PKSs. Pyrones **7** and **8** collectively represent products in which the PKS module-1 incorporates the cognate extender unit while pyrones **9** and **10** collectively represent molecules in which module-1 incorporates a noncognate extender unit. Product profiles and yields, relative to the 4,5-dichloropyrrolyl-SNAC substrate, for (**B**) P1 and P2, and (**C**) C1 and C2 PKSs.

For the P1 and P2 PKSs, only Group A pyrone products were observed (Figures 3B, S16–S25). This implies that whenever the Plt module-1 incorporated a noncognate extender unit, the strict gatekeeping activity of module-2 KS prohibited further polyketide extension to occur. From an engineering perspective, the deliverable here is that the gatekeeping activity of collinear KS domains can preclude product diversification.

We had previously noted the formation of **3** by the C1 and C2 PKSs which implies that the Cal module-2 KS domain was tolerant to noncognate extender units incorporated by the Cal module-1 (Figure 2E, 2F). In line with this observation, in the expanded competition experiment, we indeed observed the production of Group B pyrones, together with Group A pyrones, by C1 and C2 PKSs (Figure 3C). Even here, the abundance of Group A was greater than that of the Group B pyrones. FabD has been shown to be proficient for acylating CPs using Mal- and MeMal extender units.^6^ Under the conditions used for triketide pyrone production in this study, FabD was also proficient in incorporating EtMal and PgMal extender units (Figures S26–S28). This implies that the Cal module-2 KS was indeed challenged by noncognate extender unit incorporation by the Cal module-1 (albeit to a lesser degree than the Plt module-2 KS), with the PgMal-extended diketide being especially difficult in being accommodated by the module-2 KS. Using the PgMal extender unit, the production of the Group B pyrones by C1 and C2 PKSs was markedly lower, implying that the Cal module-1 *cis*-AT *and* FabD were both challenged in using PgMal-CoA as the substrate to load the Cal module-1 CP (Figure S21).

Taken together, a concerted view of KS gatekeeping emerges. A KS domain has two substrates: a ketide substrate, and an extender unit (Figure 4). The ketide substrate is transthioesterified from the CP to the KS active site, which is when the decarboxylative condensation with the extender unit occurs. Data presented herein allow us to posit that KS domains are tolerant for different extender units while being selective for their ketide substrates. For the assembly line illustrated in Figure 4, if the native extender unit is incorporated by KS_1_, then the correct ketide substrate would be presented to the downstream KS_2_, and KS_2_ will allow for further polyketide extensions to occur; KS_1_ by itself is not selective for the extender unit that it incorporates. In the event that an incorrect extender unit is added by KS_1_, an incorrect ketide substrate will be presented to KS_2_, and KS_2_ would then preclude further extensions. Thus, the downstream KS_2_ would act as a gatekeeper to check the transformation affected by upstream KS_1_. Generalized rules regarding KS specificity are difficult to derive; the KS domains for Plt and Cal PKSs are different in their substrate selectivity. It is likely that an independent evaluation KS domain activity for non-native ketide substrates and extender units would provide valuable insights into the feasibility of engineering PKS assembly lines. Our findings that the Plt KS domains are selective for their ketide substrates is perhaps a reflection of the coevolution of the KS domains with their upstream AT and CP domains.^8,9^

**Figure 4:**
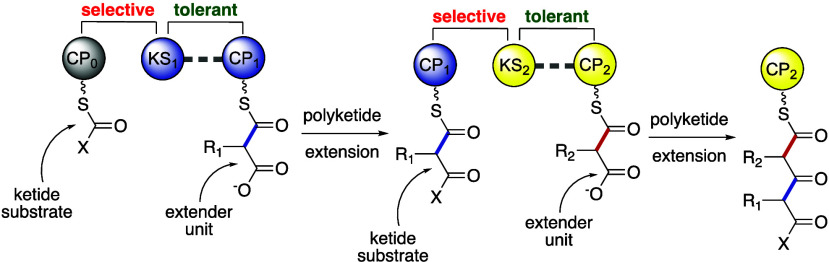
Model of how KS domains maintain fidelity in PKS assembly lines.

## Supporting Information Available

Experimental details, Figures S1–S28, and Table S1 (PDF)

## Accession Codes

The Plt and Cal PKSs are associated with the GenBank accession numbers AAC38075.1 and HM452329.1, respectively. The GenBank accession number associated with the *E. coli* FabD is CAD6016765.1.
