# Supplementary material for: Gatekeeping Activity of Collinear Ketosynthase Domains Limits Product Diversity for Engineered Type I Polyketide Synthases
Source: Biochemistry. 2024 Aug 26;63(18):2240–4. doi: 10.1021/acs.biochem.4c00249 (PMC11411704; doi:10.1021/acs.biochem.4c00249)
Supplement: Supplementary file 1 — bi4c00249_si_001.pdf [file bi4c00249_si_001.pdf]

SUPPLEMENTARY INFORMATION FOR

Gatekeeping activity of collinear ketosynthase domains limits product diversity for engineered type I polyketide synthases

Dongqi Yi,[1] Mujeeb A. Wakeel,[1] and Vinayak Agarwal[1,2,*]

[1]School of Chemistry and Biochemistry, Georgia Institute of Technology, Atlanta, GA 30332, USA

[2]School of Biological Sciences, Georgia Institute of Technology, Atlanta, GA 30332, USA

*Correspondence: vagarwal@gatech.edu; Ph: (+1)404-385-3798

SUPPLEMENTARY MATERIALS AND METHODS

General materials and instrumentation

All chemicals, solvents, and media components were obtained commercially from Sigma-Aldrich, Fisher Scientific, and VWR, and used without further purification. All enzymes were purchased from New England Biolabs or Takara Bio. Reactions were monitored by thin layer chromatography (TLC) carried out on Supelco silica gel (60 F_{254}) glass plates using UV light as the visualizing agent, and basic aqueous $KMnO_4$ with heat as developing agents. Nuclear magnetic resonance (NMR) spectra were recorded on Bruker Avance III HD 500 MHz instruments and calibrated using residual undeuterated solvent as the internal reference (MeOD δ_H 3.31 and δ_C 49.00). The splitting patterns were reported as s=singlet, d=doublet, t=triplet.

Cloning, protein expression and protein purification

The construction of expression vectors for Plt module-1 with DEBS2 C-terminal docking domain (dd) sequences, CalA module-1 with DEBS2dd, PltB module-2 with DEBS3 N-terminal docking domain sequences, MatB, Sfp and FabD has been described previously.[1] Based on the primary protein sequence, the domain boundary of the Cal module-2 was mapped to residues 1073 through 2942 of the CalA polypeptide. To inactive the Cal module-2 KR domain, residue Y2677 was replaced with phenylalanine. Similar to Plt module-2, the N-terminal of Cal module-2 was fused to DEBS3dd sequence. Due to the large size of CalA module-2 sequences (5610 bp), the corresponding gene sequence was divided evenly into two DNA fragments and synthesized by Twist Biosciences. The two Cal module-2 DNA fragments were amplified using the synthesized DNA as template with PrimeSTAR high-fidelity DNA polymerase in PCR reactions, and the PCR amplicons were assembled into pET28(+) vector using Gibson assembly. The sequence of the recombinant vector was confirmed by nanopore sequencing (Plasmidsaurus).

For overexpression of holo-PKSs, the kanamycin-resistant pET28(+) vector carrying the PKS modules and the streptomycin-resistant pCDFDuet-1 vector carrying Sfp were co-transformed into *E. coli* BL21Gold(DE3). Overnight culture was inoculated in 2 or 4 L terrific both media supplemented with kanamycin (50 mg/L) and streptomycin (50 mg/L), and grown at 30 ˚C. When OD_{600} reached 0.4–0.5, the culture temperature was reduced to 18 ˚C. When OD_{600} reached 0.7–0.8, protein expression was induced by the addition of 0.1 mM IPTG together with 0.25 mM calcium pantothenate. Cells were cultured at 18 ˚C for an addition of 18 h before being harvested by centrifugation. Cell pellets were resuspended in binding buffer (50 mM potassium phosphate pH=7.6, 10% glycerol) and stored at –80 ˚C until protein purification. All protein purification steps were performed on ice or at 4 ˚C. Cell pellets were thawed and lysed by

sonication. The lysate was clarified by centrifugation at 27,000×g for 45 min, and the supernatant was applied to a 5 mL HisTrap HP column. The column was washed with 10 column volumes of wash buffer (50 mM potassium phosphate pH=7.6, 30 mM imidazole, 50 mM NaCl, 10% glycerol), and then eluted with a linear gradient to 100% of elution buffer (50 mM potassium phosphate pH=7.6, 250 mM imidazole, 50 mM NaCl, 10% glycerol) over 8 column volumes using ÄKTAprime plus FPLC system. Purity of eluent protein fractions was checked by SDS-PAGE, and fractions containing desired purified proteins were combined. The combined protein solution was either applied to a 5 mL HiTrap Q HP column or directly concentrated using spin centrifugal filters. For anion exchange, after loading of protein solutions, the column was washed with 5 column volumes of buffer A (50 mM potassium phosphate pH=7.6, 50 mM NaCl, 10% glycerol), and then eluted with buffer B (50 mM potassium phosphate pH=7.6, 600 mM NaCl, 10% glycerol). For protein concentration, pooled protein solutions were concentrated using 50 kDa Amicon centrifugal filters and desalted into buffer B with PD-10 columns. Purified proteins were stored in small aliquots at –80 ˚C. Fresh aliquots were used each time for enzyme assays.

For MatB, FabD, and *E. coli* CP, the expression vectors carrying genes of interest were transformed into *E. coli* BL21Gold(DE3). Similar growth condition to PKS modules was used, except that 1 L terrific broth medium was used for cell culture and protein expression was induced with 0.2 mM IPTG. In addition, similar protocols were used for protein purification. Cell pellets were resuspended in binding buffer (20 mM Tris-HCl pH=8.0, 500 mM NaCl, 10% glycerol) and lysed by sonication. The lysate was clarified by centrifugation and applied to a 5 mL HisTrap HP column. The column was washed with 10 column volumes of wash buffer (20 mM Tris-HCl pH=8.0, 30 mM imidazole, 500 mM NaCl, 10% glycerol), and then eluted with a linear gradient to 100% of elution buffer (20 mM Tris-HCl pH=8.0, 250 mM imidazole, 500 mM NaCl, 10% glycerol) over 8 column volumes. For MatB, protein solutions were concentrated using 30 kDa Amicon centrifugal filters and desalted into binding buffer with PD-10 columns. FabD and *E. coli* CP protein solutions were dialyzed overnight in 2 L binding buffer before storage. Purified proteins were stored in small aliquots at –80 ˚C. Fresh aliquots were used each time.

Enzymatic preparation of acyl-CoAs

The MatB-catalyzed production of Mal-, MeMal-, EtMal-, and PgMal-CoAs has been described previously.[2] In brief, reactions were performed in a total volume of 150 µL comprising 2.5 mM CoA-SH, 5 mM TCEP, 5 mM malonate or other malonyl analogs, 5 mM ATP, 10 mM $MgCl_2$, 15% glycerol, 400 mM potassium phosphate (pH = 7.5) and 10 µM purified MatB. The reaction mixtures were incubated at 30 ˚C for 16 h, and frozen at –80 ˚C until future use in PKS assays. Complete conversion of CoA-SH was confirmed by HPLC using Agilent 1260 Infinity II HPLC system with Phenomenex Luna 5 µm C8(2) 100

Å LC column (250×4.6 mm). Water (solvent A) and MeOH (solvent B) with 0.1% TFA were used as the mobile phase. A flow rate of 0.5 mL·min^{-1} was used with the following gradient: 0–5 min: 5% B, 5–30 min: linear gradient to 80% B, 30–34 min: linear gradient to 100% B, 34–38 min: 100% B, 38–39 min: linear gradient to 5% B, 39–40 min: 5 % B, 40-41 min: linear gradient to 100% B, 41–42 min: 100% B, 42–43 min: linear gradient to 5% B, 43-45 min: 5%. UV absorbance was monitored at 254 and 280 nm.

Enzyme assays for triketide pyrone formation

PKS assays for triketide pyrone product formation were performed in a total volume of 100 µL containing 50 µM 4,5-dichloropyrrolyl-SNAC, 0.5 mM Mal- or MeMal-CoA, 400 mM potassium phosphate buffer (pH=7.5), 5 µM module-1 and module-2 PKSs, and optionally, 5 µM FabD. The competition assays comprised of 50 µM 4,5-dichloropyrrolyl-SNAC, 0.5 mM Mal- or MeMal-CoA, 0.5 mM other extender units, 400 mM potassium phosphate buffer (pH=7.5), 5 µM module-1 and module-2 PKSs, and optionally, 5 µM FabD in a total volume of 100 µL. The reactions were incubated at 30 ˚C for 16 h, before being quenched by the addition of 50 µL MeOH containing 10% v/v formic acid. Production of pyrone products was monitored by LC/MS and HPLC.

HPLC analyses to quantify the yield of pyrone products were carried out using Phenomenex Luna 5 µm C8(2) 100 Å LC column (4.6×250 mm) using Agilent 1260 Infinity II HPLC system. Water (solvent A) and MeCN (solvent B) with 0.1 % TFA were used as the mobile phase at a flow rate of 0.5 mL·min^{-1} with the following gradient: 0–5 min: 5% B, 5–30 min: linear gradient to 100% B, 30–34 min: 100% B, 34–35 min: linear gradient to 5% B, 35–36 min: 5 % B, 36–37 min: linear gradient to 100% B, 37–38 min: 100% B, 38–39 min: linear gradient to 5% B. For analyses of pyrones with a propargyl group, the following gradient was used: 0–5 min: 5% B, 5–22 min: linear gradient to 55% B, 22–36 min: 55% B, 36–40 min: linear gradient to 100% B, 40-44 min: 100%, 44–45 min: linear gradient to 5 % B, 45–47 min: 5% B. UV absorbance was monitored at 352 nm. Peak area at 352 nm was converted to the amount of pyrone formed in the assay using a standard curve of **1**.

LC/MS analyses were carried out using Agilent Poroshell 120 EC-C_{18} column (2.7 µm, 4.6×100 mm) using Agilent 1260 Infinity HPLC coupled to a Bruker amaZon SL mass spectrometer. Data were acquired in the negative ionization mode. Water (solvent A) and MeCN (solvent B) with 0.1 % formic acid were used as the mobile phase. A flow rate of 0.5 mL·min^{-1} was used with the following gradient (except for propargyl pyrones): 0–3 min: 5% B, 3–16 min: linear gradient to 100% B, 16–20 min: 100% B, 20–21 min: linear gradient to 5% B, 21–22 min: 5% B, 22–23 min: linear gradient to 100% B, 23–24 min: 100% B, 24–25 min: linear gradient to 5% B, 25–27 min: 5% B. For analyses of pyrones with a propargyl group,

the following gradient was used: 0–3 min: 5% B, 3–11 min: linear gradient to 55% B, 11–18 min: 55% B, 18–22 min: linear gradient to 100% B, 22-26 min: 100%, 26–27 min: linear gradient to 5 % B.

Single module competition assays

Single module competitive assays were performed in a total volume of 70 µL containing 20 µM 4,5-dichloropyrrolyl-SNAC, 20 µM module-1 PKSs, 0.2 mM Mal-CoA, 0.2 mM MeMal-CoA, and 400 mM potassium phosphate buffer (pH=7.5). The reactions were incubated at 30 ˚C for 1 h before being quenched with 30 µL MeOH containing 23% v/v formic acid to adjust pH to 2. For the time-course assay, the assays were performed in a total volume of 480 µL with the same components as the end-point assay. At time points 5 min, 15 min, 30 min, 60 min, and 90 min, 70 µL samples were withdrawn and quenched as before. The quenched reactions were centrifuged and analyzed by LC/MS using the same method as described above. Ions corresponding to $[M\text{-}CO_2\text{-}H]^{1-}$ *m/z* of elongated diketides were extracted, and peak areas for extracted ion counts were integrated. To represent the percentage of a diketide product in the competitive assay, the ratio of its EIC peak area to the summed EIC peak areas of all diketide products was calculated.

Synthesis of 2-propargylmalonic acid (S1)

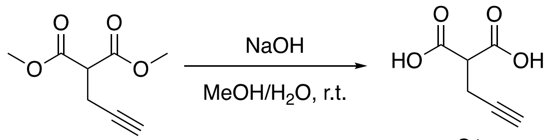

S1

The molecule **S1** was prepared as previously described and the ^{1}H and ^{13}C NMR spectra acquired in this study match those reported in the literature.[3] To a stirring solution of dimethyl propargylmalonate (1 g, 0.89 mL, 5.88 mmol) in 25 mL MeOH, 24 mL 1 M NaOH aqueous solution was added slowly. The reaction was stirred at room temperature for 1 h and monitored by TLC. The reaction was quenched by the addition of 1 M HCl to adjust pH to 2, and extracted with EtOAc (50 mL, 3×). The organic layers were combined, dried with anhydrous Na_2SO_4, and concentrated under vacuum to give **S1** as a white solid (577.8 mg, 69%). ^{1}H NMR (500 MHz, MeOD) δ 3.51 (td, J = 7.6, 1.1 Hz, 1H), 2.70 (dd, J = 7.6, 2.7 Hz, 2H), 2.32 (t, J = 2.7 Hz, 1H). ^{13}C NMR (126 MHz, MeOD) δ 171.49, 81.38, 71.21, 52.55, 19.26. HRMS (ESI) *m/z* calculated for $C_6H_5O_4$ ($[M–H]^{1–}$) 141.0193, found 141.0194.

SUPPLEMENTARY TABLES

Table S1: Extracted ion count peak areas of diketide formation in single-module competitive assay averaged from three replicate experiments

**PKS system**	**EIC peak areas**	
	Diketide **5**	Diketide **6**
**P1**	17035921	38462057
**P2**	41316299	39904385
**C1**	9998226	40208893
**C2**	35342240	64359916

SUPPLEMENTARY FIGURES

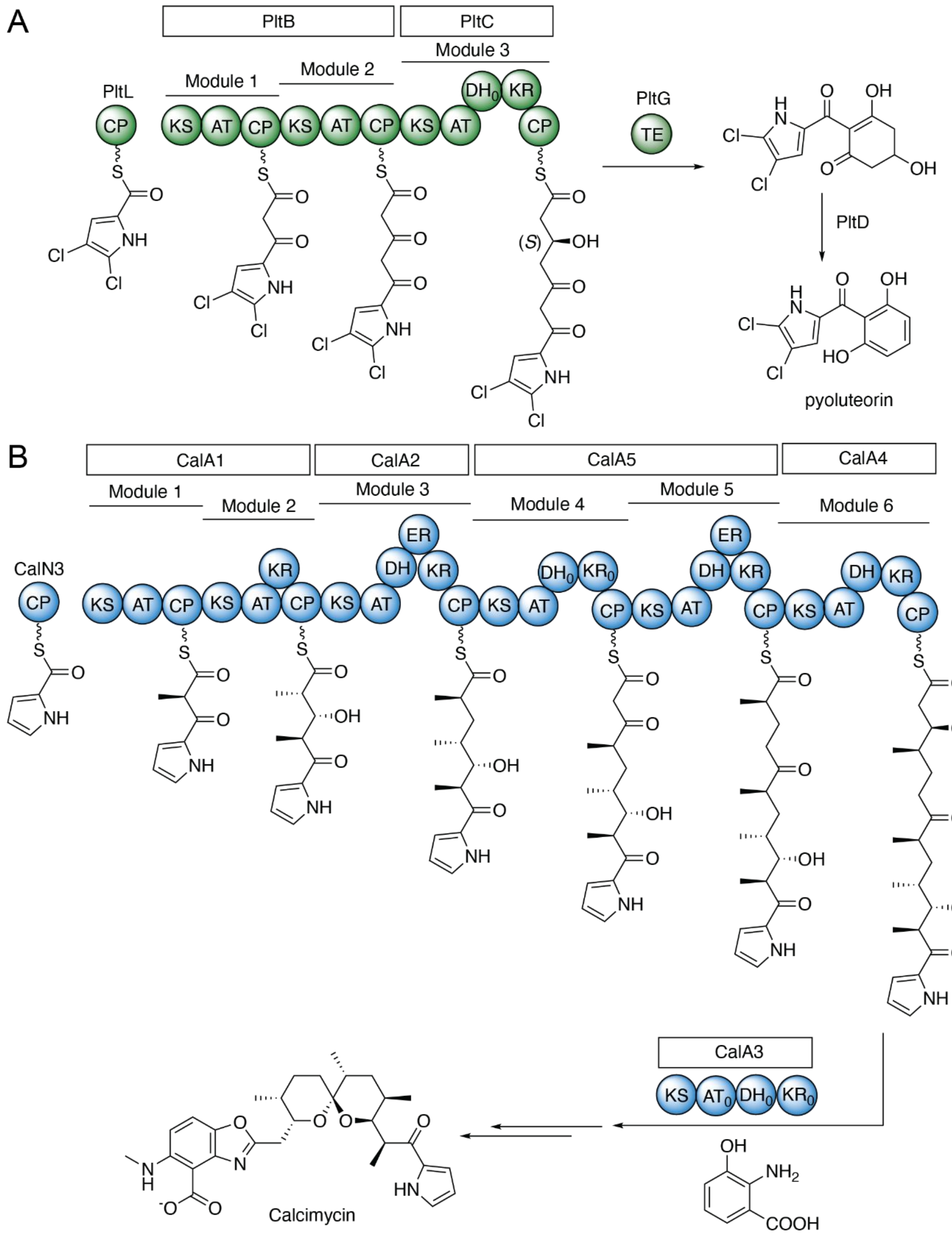

Figure S1: (**A**) The pyoluteorin (Plt) and (**B**) calcimycin (Cal) assembly lines. Note that the Plt module-2 bears a non-functional KR domain. The Cal module-2 bears a functional KR domain which was inactivated by active site mutagenesis in this study.

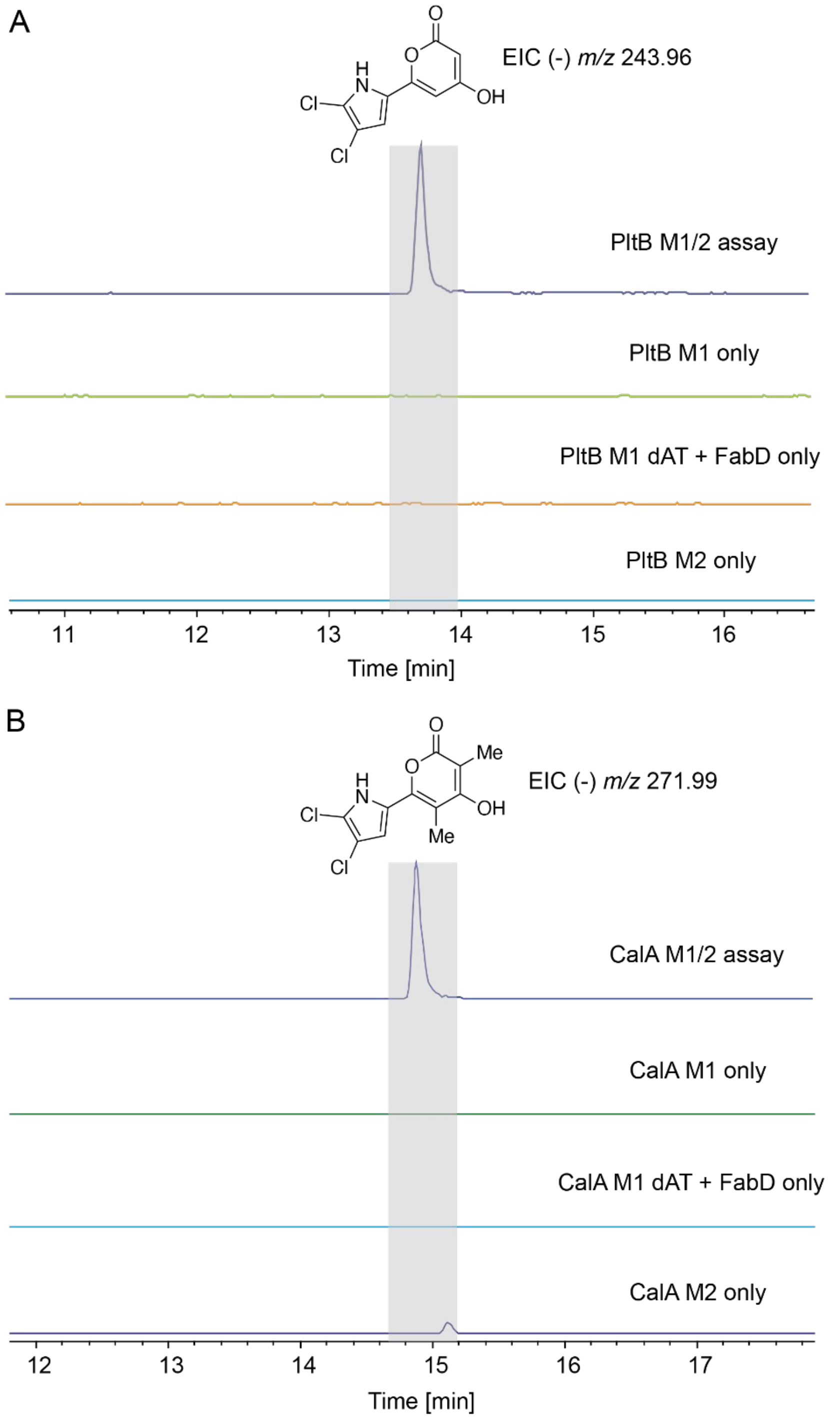

Figure S2: Extracted ion chromatograms demonstrating the production of pyrone **1** ([M-H]$^{1-}$ *m/z* 243.96 Da) in Plt (**A**) and **2** ([M-H]$^{1-}$ *m/z* 271.99 Da) in Cal (**B**) PKS assays with their native extender units. Pyrone formation was observed when both PKS modules were provided in the assay, while no or trace amount of pyrone was detected with only module-1 or module-2, which indicates that the production of pyrone products is not due to the iterative activity of PKSs. Here, 'dAT' refers to inactivated AT domain.

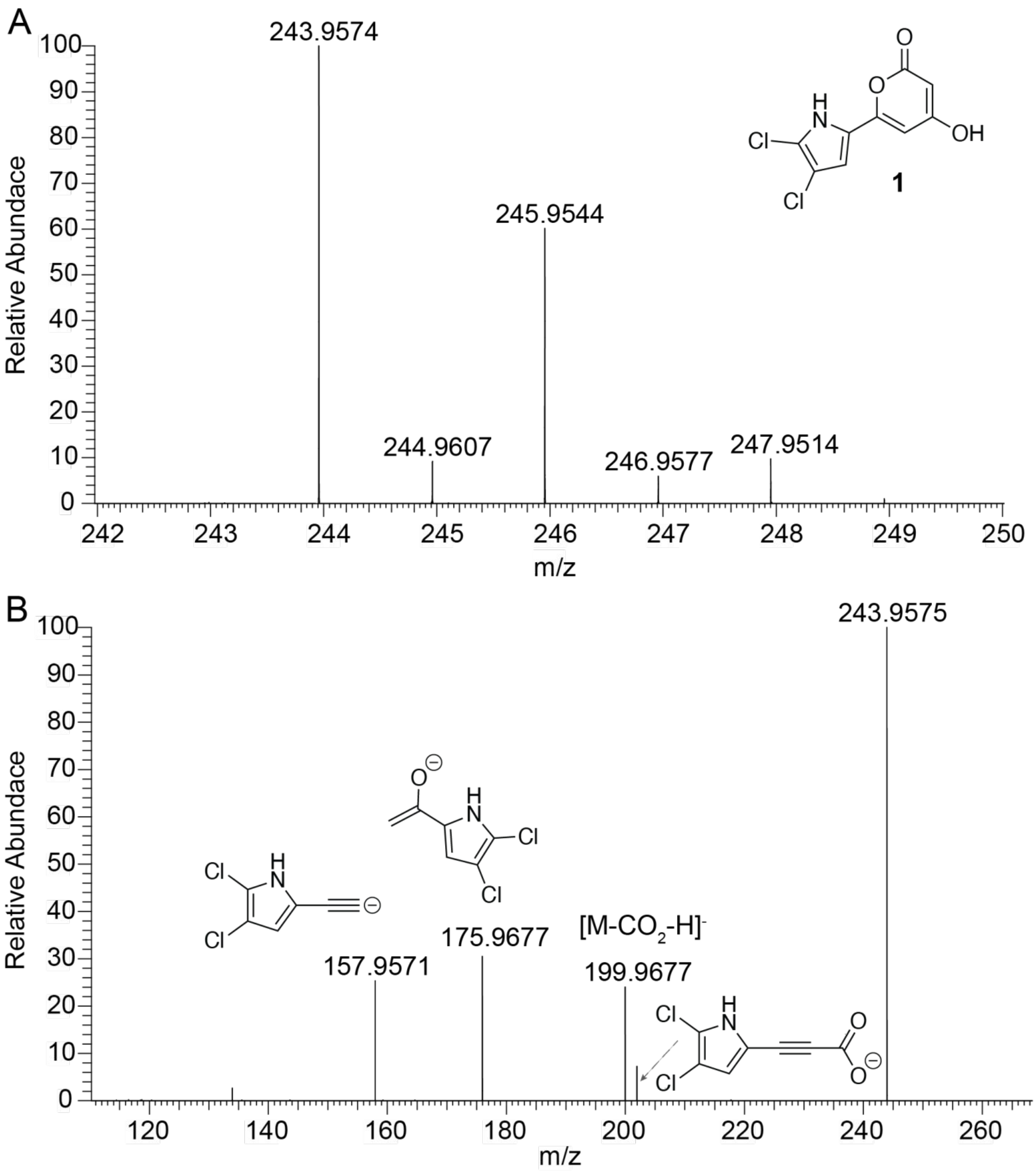

Figure S3: MS^1 and MS^2 spectra of **1**. (**A**) HRMS (ESI) identified molecule ions corresponding to $[M–H]^{1–}$ for compound **1** (*m/z* calculated for $C_9H_4Cl_2NO_3$ 243.9574, found 243.9575). (**B**) MS^2 spectra of compound **1** with rationalized structural annotations of fragment ions.

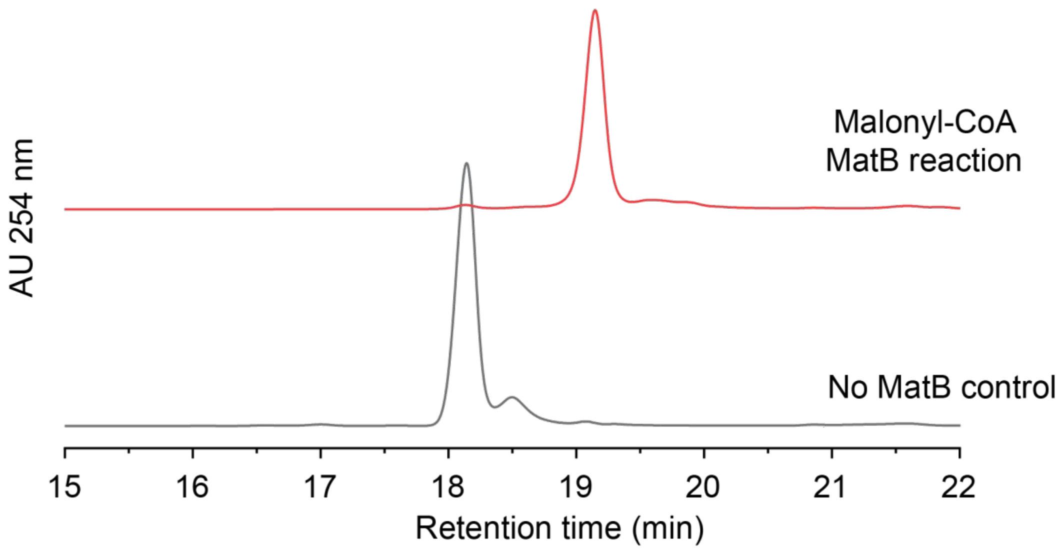

Figure S4: MatB-catalyzed production of Mal-CoA. Compared to negative control, CoA-SH was completely converted to Mal-CoA products in enzyme assays.

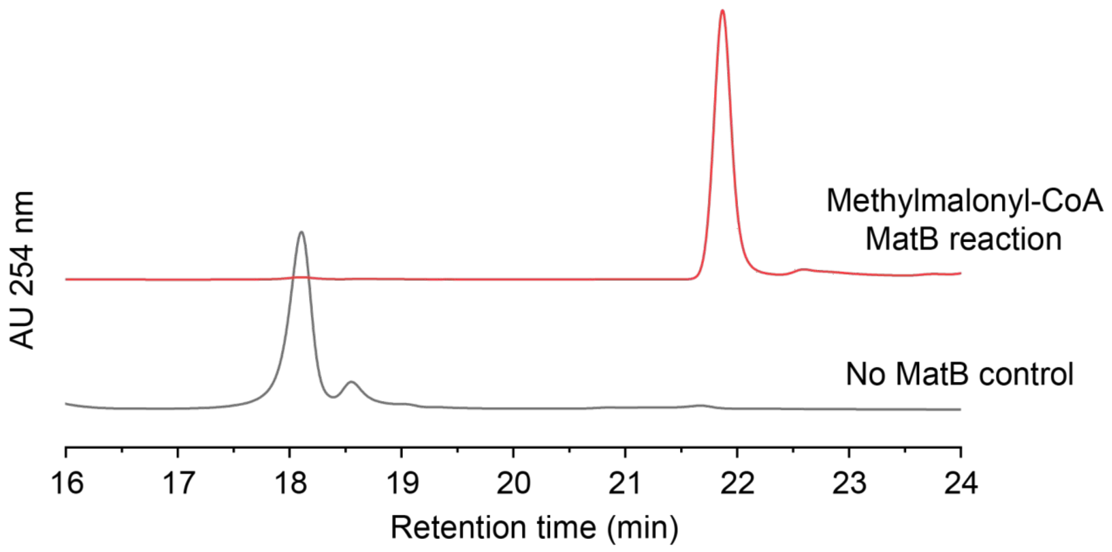

Figure S5: MatB-catalyzed production of MeMal-CoA. Compared to negative control, CoA-SH was completely converted to MeMal-CoA products in enzyme assays.

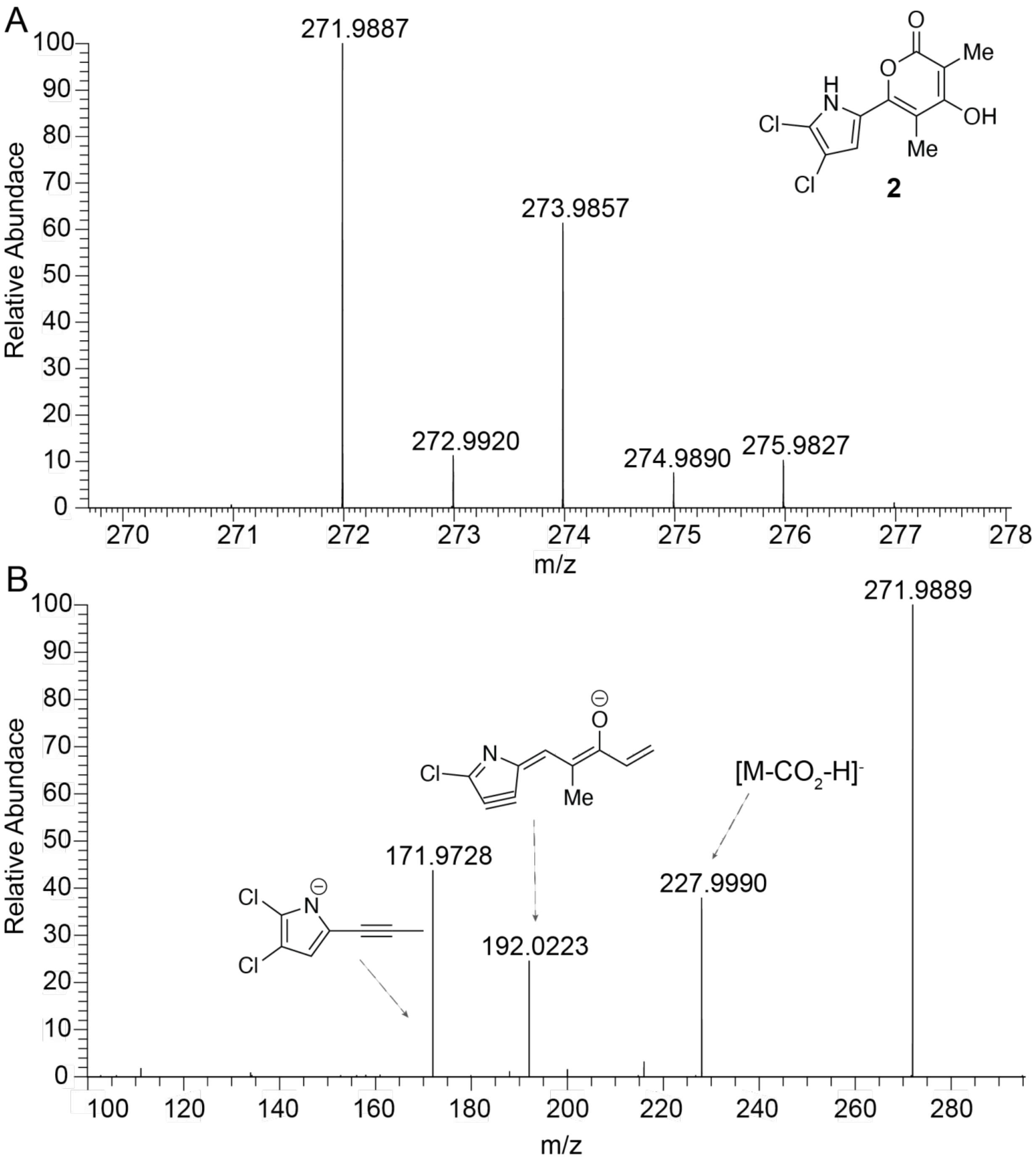

Figure S6: MS^1 and MS^2 spectra of **2**. (**A**) HRMS (ESI) identified molecule ions corresponding to $[M\text{-}H]^-$ for compound **2** (*m/z* calculated for $C_{11}H_8Cl_2NO_3$ ($[M\text{-}H]^-$) 271.9887, found 271.9887). (**B**) MS^2 spectra of compound **2** with rationalized structural annotations of fragment ions.

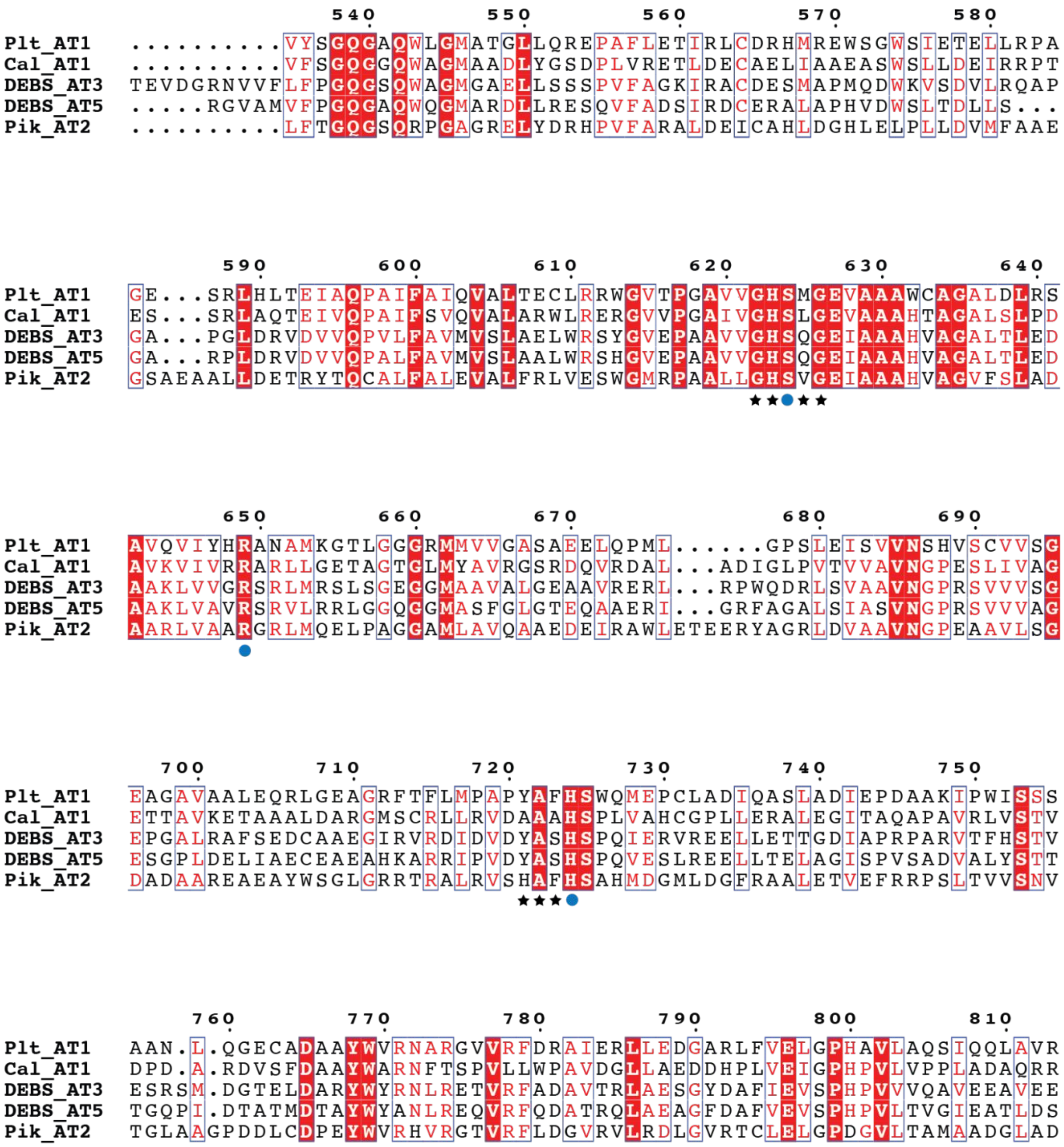

Figure S7: Sequence alignments of AT domains. Residues with more than 70% similarity were colored in red and framed in blue. The figure was generated by ESPript 3.0. Catalytic dyad Ser-His residues and the conserved Arg are highlighted by blue round circles. The characterized GHSXG and YASH/HAFH motifs are highlighted by black stars. Active site serine in Plt and Cal AT1 was mutated to alanine to generate P2 and C2 PKS systems.

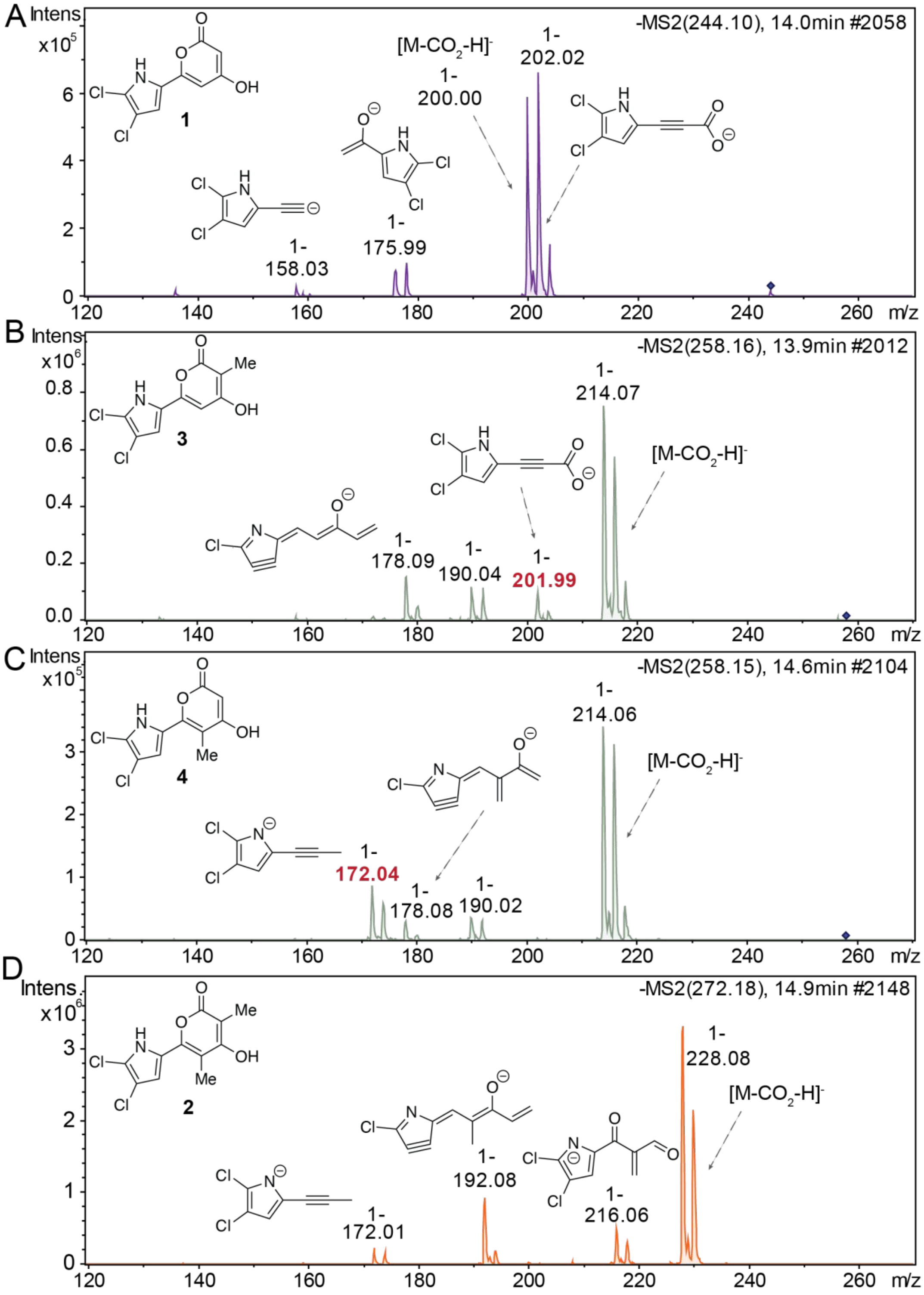

Figure S8: MS^2 spectra of pyrones **1** (**A**), **3** (**B**), **4** (**C**), and **2** (**D**) with rationalized structural annotations of fragment ions illustrated. Ions highlighted in red represented fragment ions that only exist in one isomer, but not in the other one, which enables differentiation between isomers **3** and **4**.

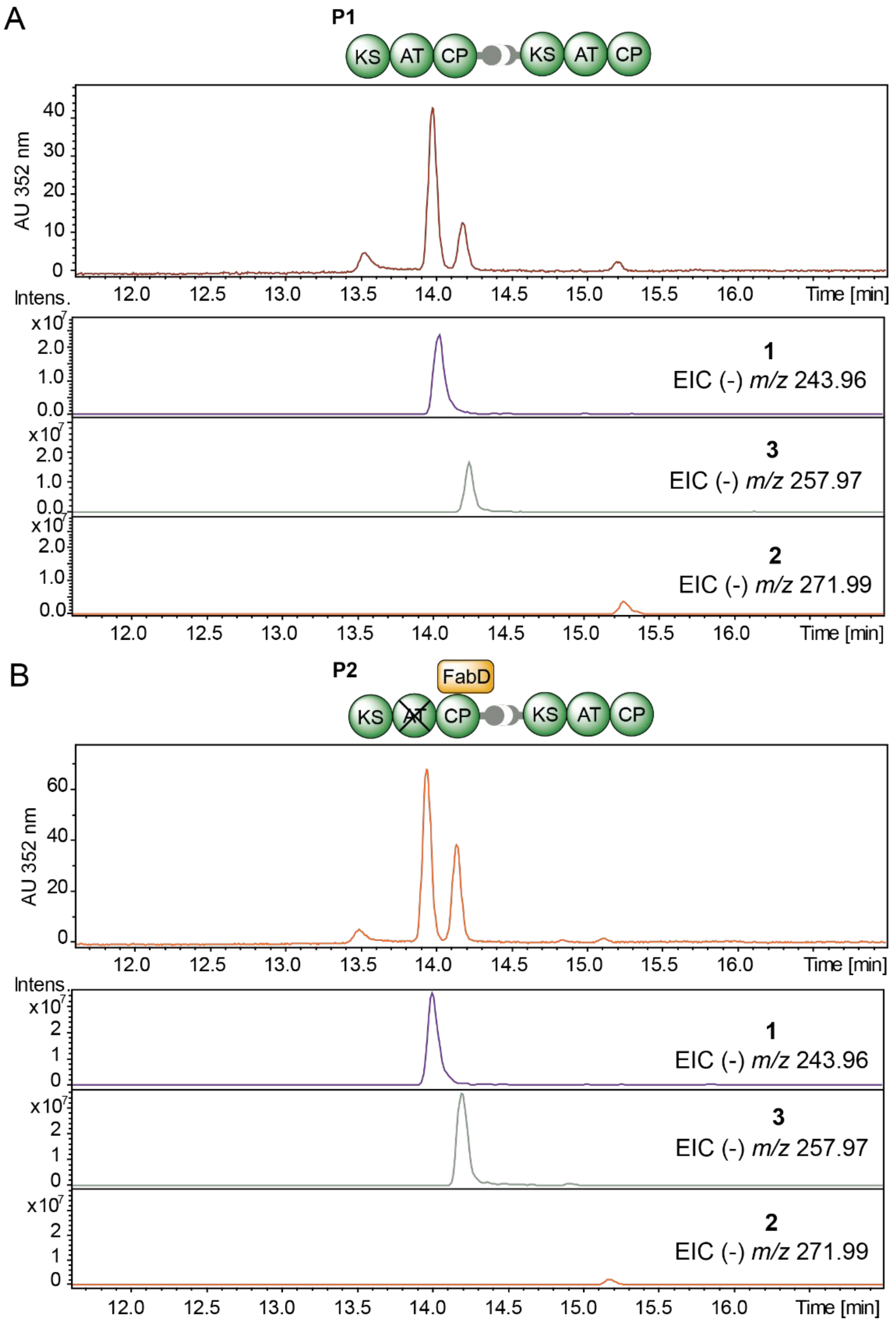

Figure S9: Detection of pyrone formation in P1 (**A**) and P2 (**B**) Plt PKS systems by LC/MS. Absorption at UV 352 nm was used to monitor the production level of pyrone molecules. Extracted ion chromatograms (EICs) corresponding to *m/z* 243.96 Da for molecule **1**, *m/z* 271.99 Da for molecule **2**, and *m/z* 257.97 Da for molecule **3** or **4** were shown to demonstrate the identity of each peak.

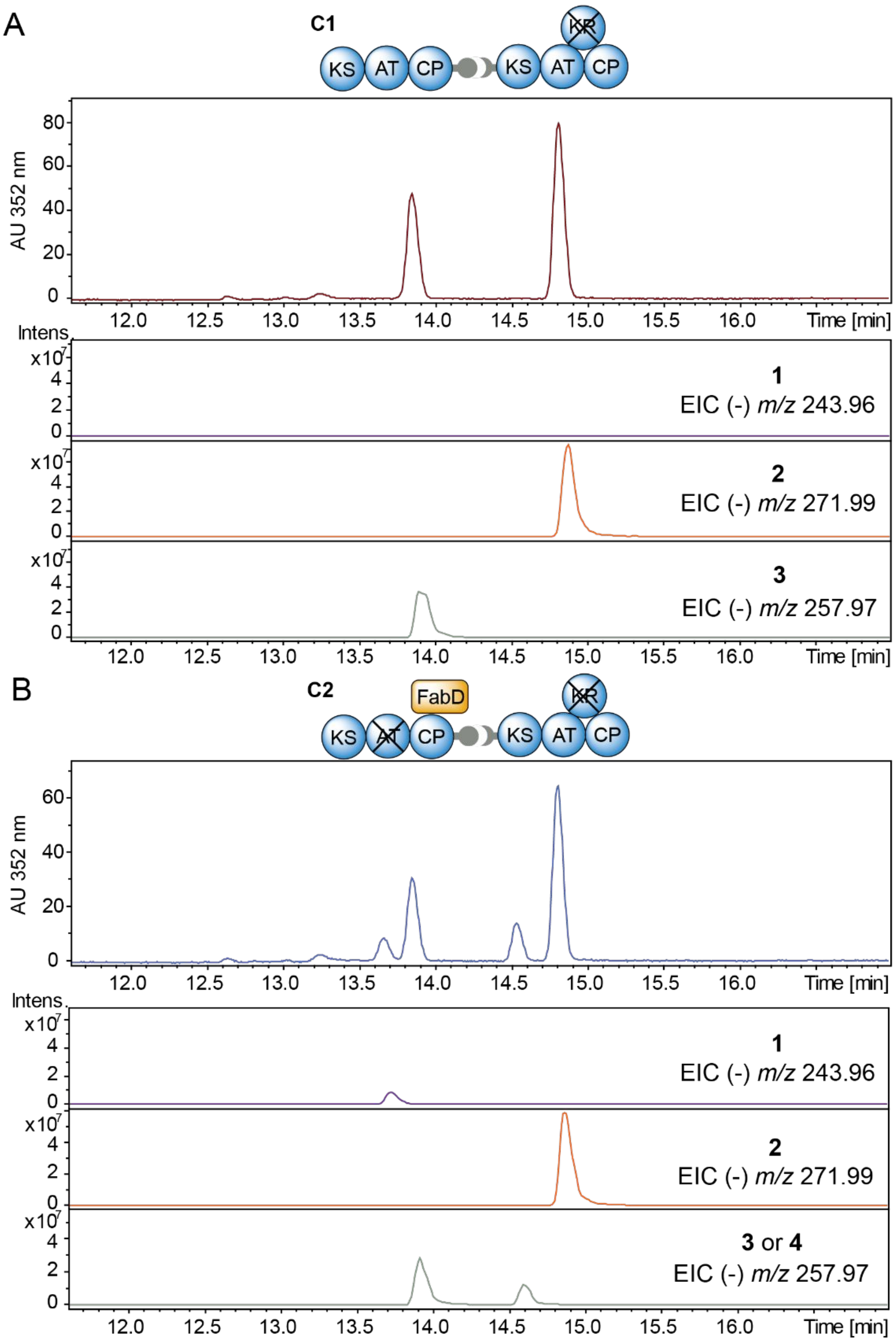

Figure S10: Detection of pyrone formation in C1 (**A**) and C2 (**B**) Cal PKS systems by LC/MS. Absorption at UV 352 nm was used to monitor the production level of pyrone molecules. Extracted ion chromatograms (EICs) corresponding to *m/z* 243.96 Da for molecule **1**, *m/z* 271.99 Da for molecule **2**, and *m/z* 257.97 Da for molecule **3** or **4** were shown to demonstrate the identity of each peak.

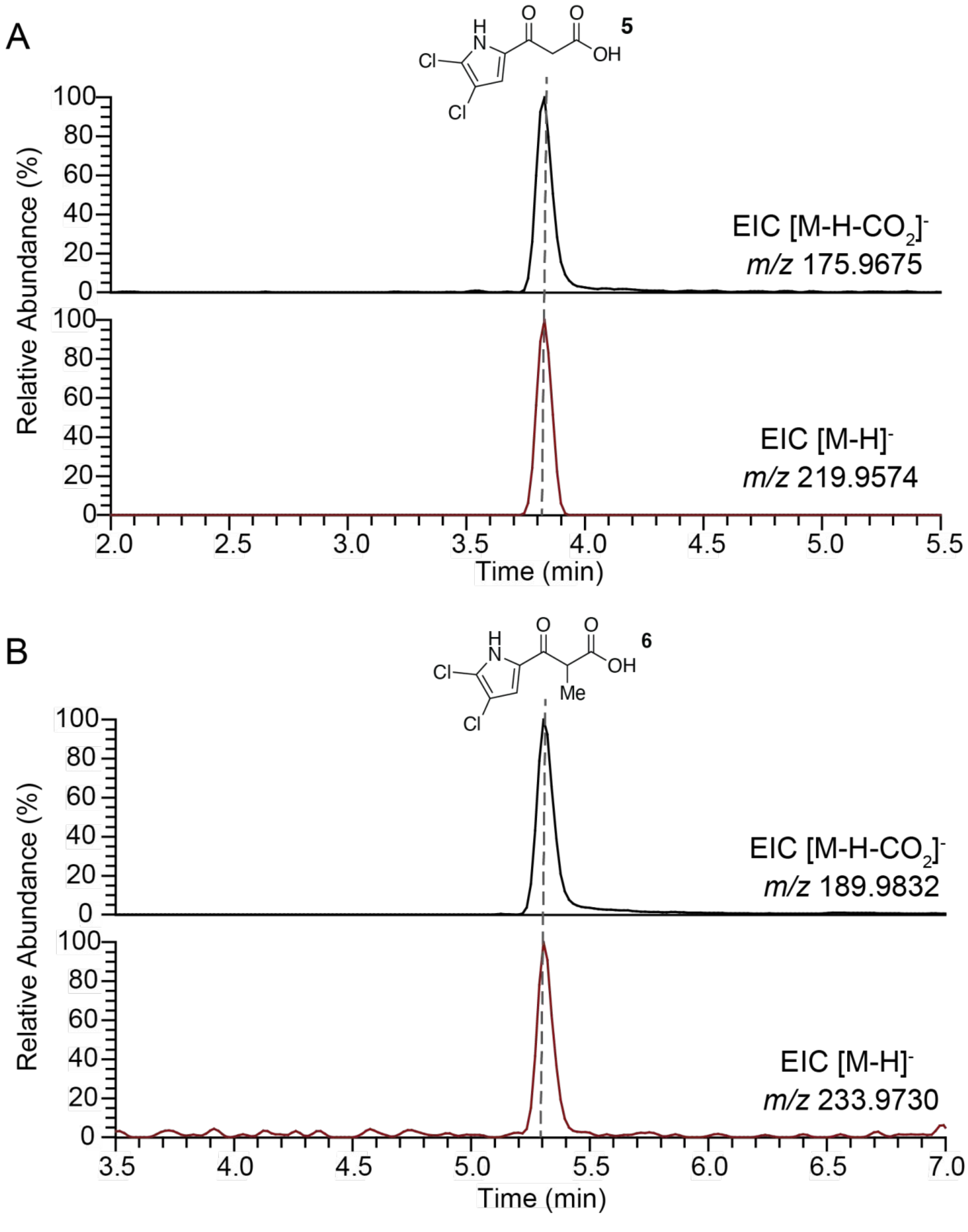

Figure S11: Production of diketides **5** and **6** in the single-module competition assay. (**A**) EICs corresponding to [M–H]$^{1-}$ *m/z* 219.9574 Da and [M–H–CO_2]$^{1-}$ *m/z* 175.9675 for diketide **5** were shown. The same retention time was observed for these peaks. (**B**) EICs corresponding to [M–H]$^{1-}$ *m/z* 189.9832 Da and [M–H–CO_2]$^{1-}$ *m/z* 233.9730 for diketide **6** were shown. The same retention time was observed for these peaks. Peak areas of extract ions for [M–H–CO_2]$^{1-}$ were used for quantifications.

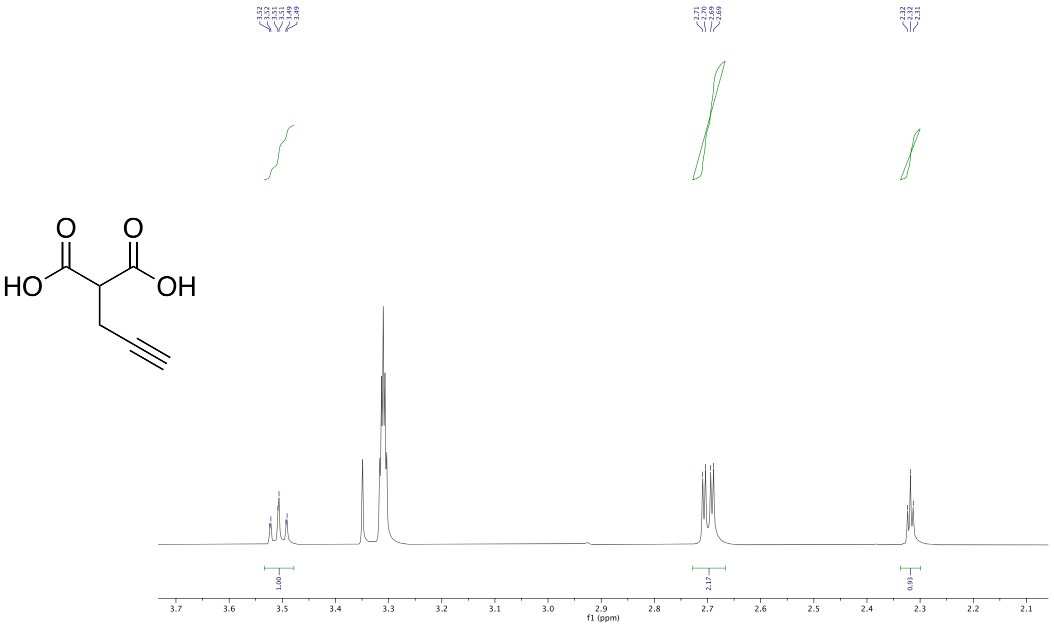

Figure S12: ^{1}H NMR spectrum (500 MHz, MeOD) of propargyl malonic acid, precursor for PgMal-CoA.

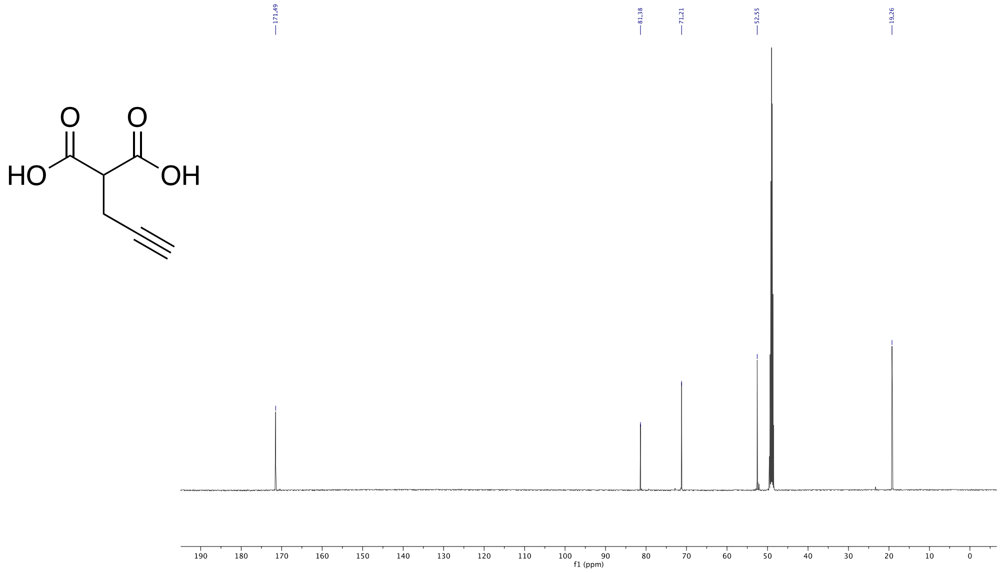

Figure S13: ^{13}C NMR spectrum (126 MHz, MeOD) of propargyl malonic acid, precursor for PgMal-CoA.

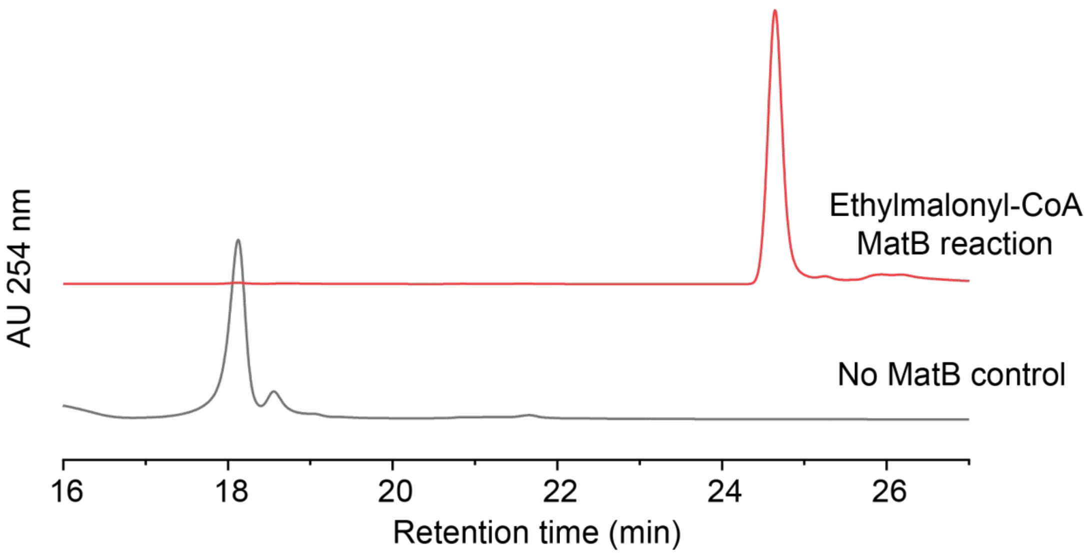

Figure S14: MatB-catalyzed production of EtMal-CoA. Compared to negative control, CoA-SH was completely converted to EtMal-CoA products in enzyme assays.

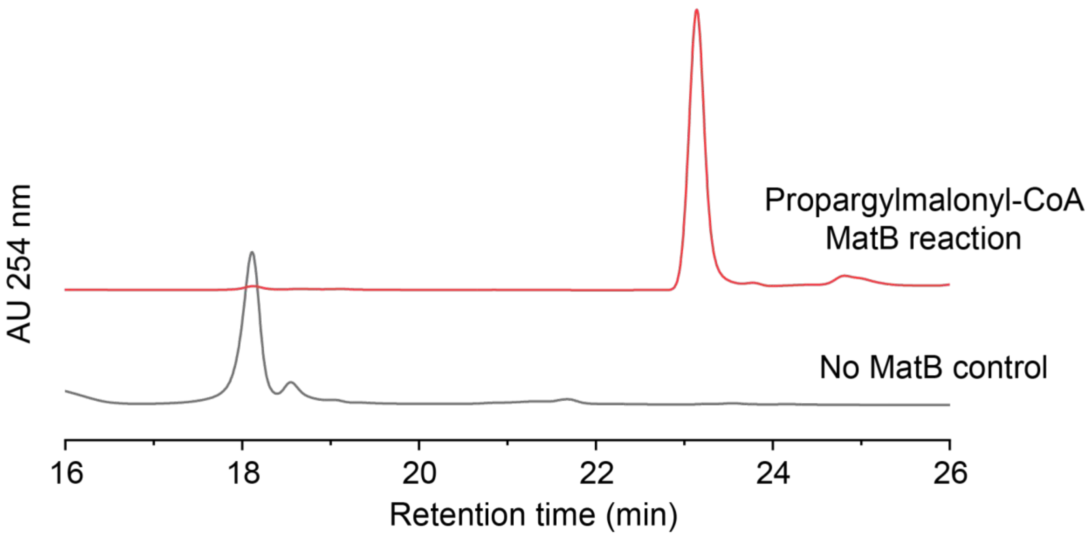

Figure S15: MatB-catalyzed production of PgMal-CoA. Compared to negative control, CoA-SH was completely converted to PgMal-CoA products in enzyme assays.

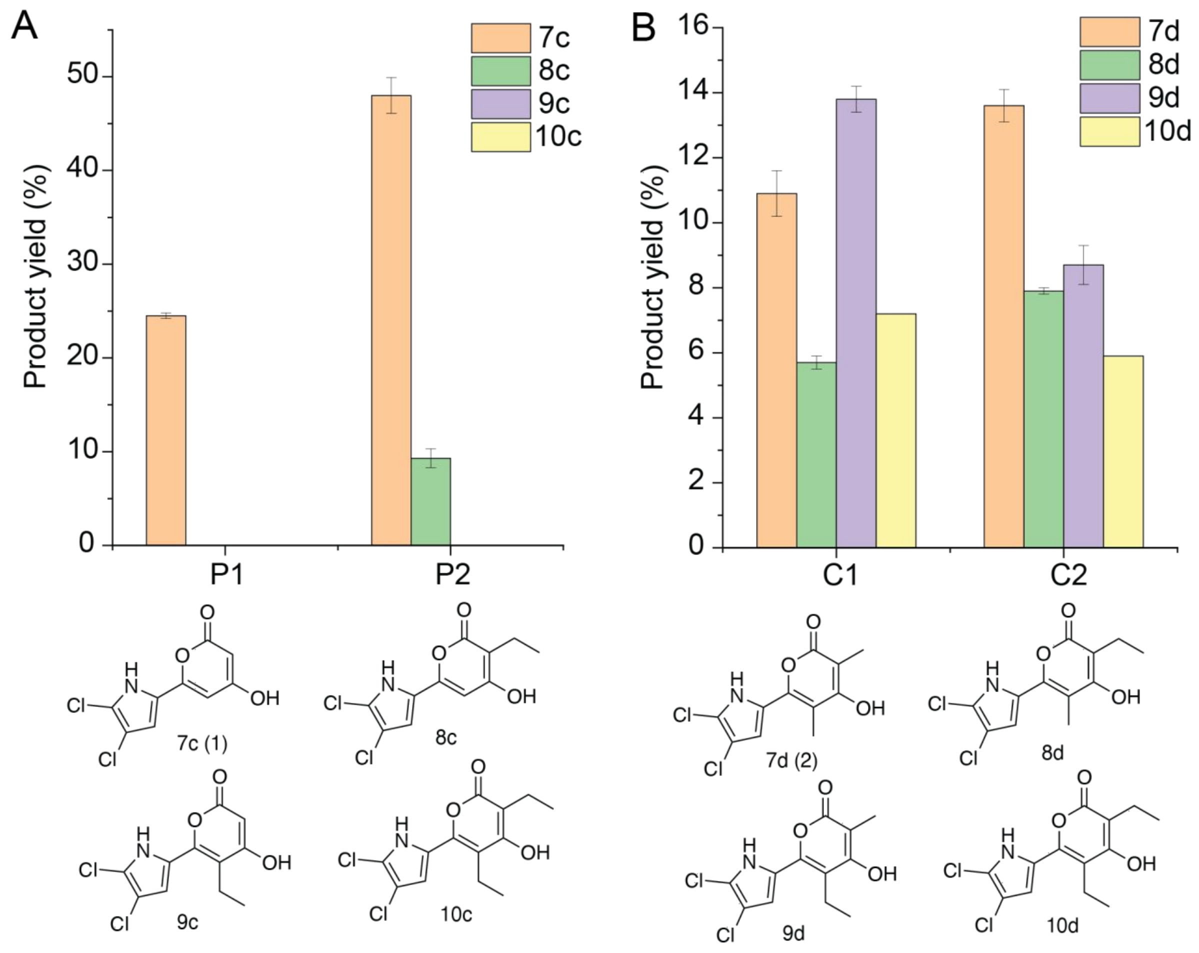

Figure S16: Product yield in the competition assay when EtMal-CoA was supplied as the non-native extender unit. The pyrone products are numbered, as illustrated. (**A**) Production of pyrones **7c–10c** by P1 and P2 PKSs. Here, **7c** and **8c** comprise of 'Group A' products, as referred to in the main text and Figure 3, and **9c** and **10c** comprise the 'Group B' products. (**B**) Production of pyrones **7d–10d** by C1 and C2 PKSs. Here, **7d** and **8d** comprise of 'Group A' products, and **9d** and **10d** comprise the 'Group B' products.

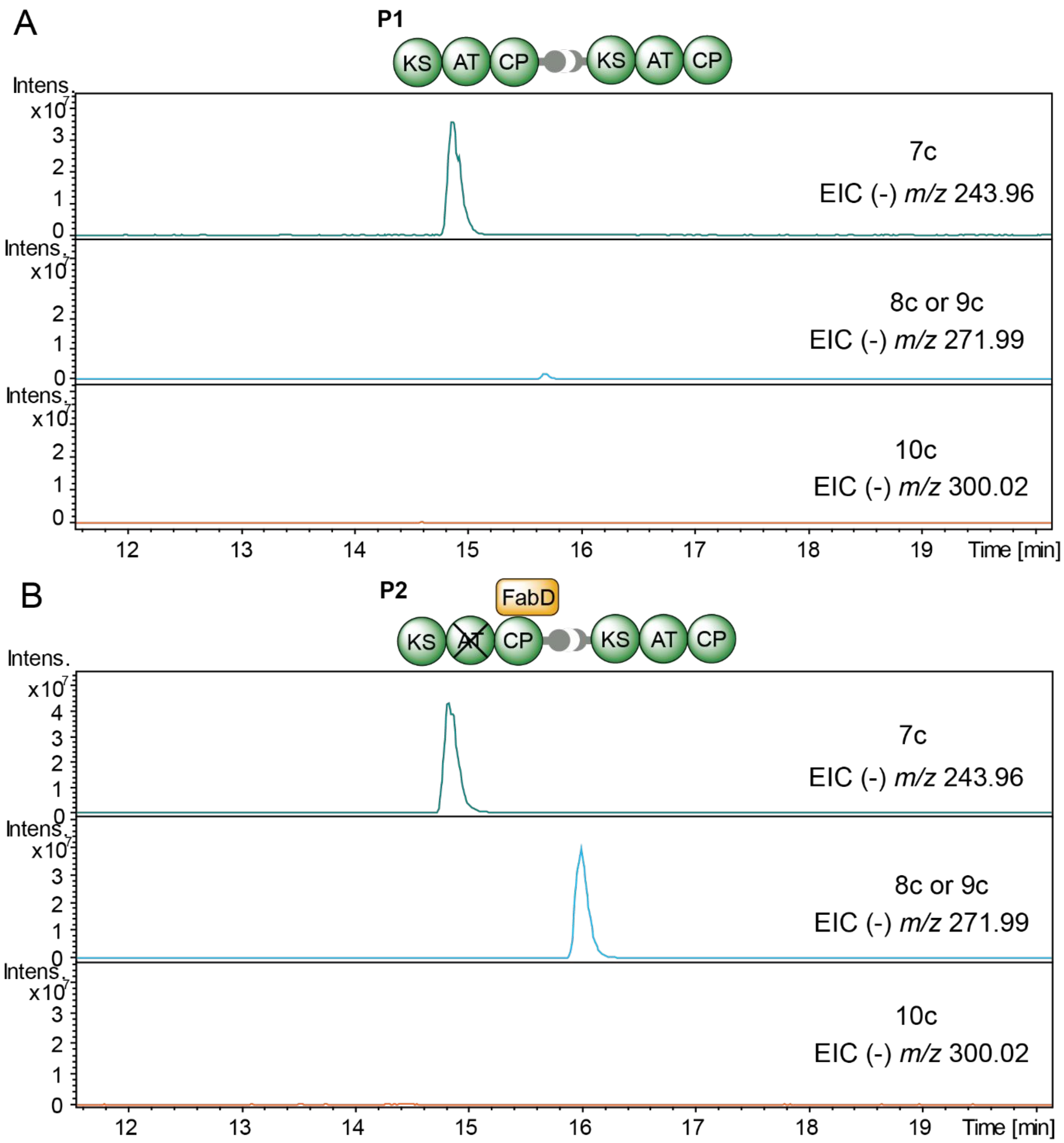

Figure S17: LC/MS-based detection of molecules **7c**–**10c** in P1 (**A**) and P2 (**B**) competition assays: **7c** is identified by EIC corresponding to $[M{-}H]^{1-}$ *m/z* 243.96 Da, **8c** and **9c** are identified by EIC corresponding to *m/z* 271.99 Da, and **10c** as identified by EIC corresponding to *m/z* 300.02 Da. Differentiating between **8c** and **9c** was enabled by their MS^2 fragmentation spectra as shown in Figure S18. Production of compound **10c** was not detected in either P1 or P2 Plt system.

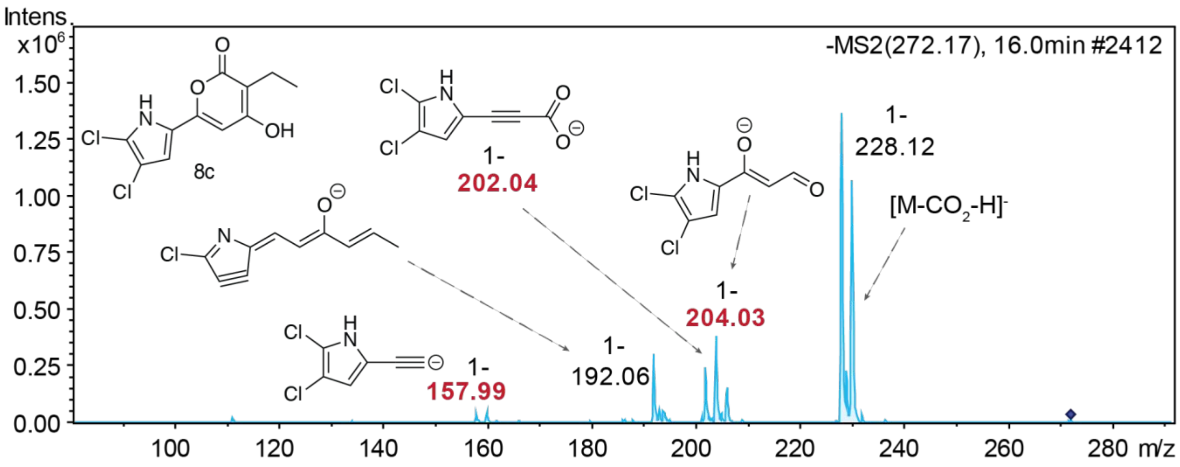

Figure S18: MS^2 spectra of **8c** with rationalized structural annotation of fragment ions. The presence of fragment ions highlighted in red demonstrated that the incorporation of the ethyl group occurred in the second elongation event to produce **8c**.

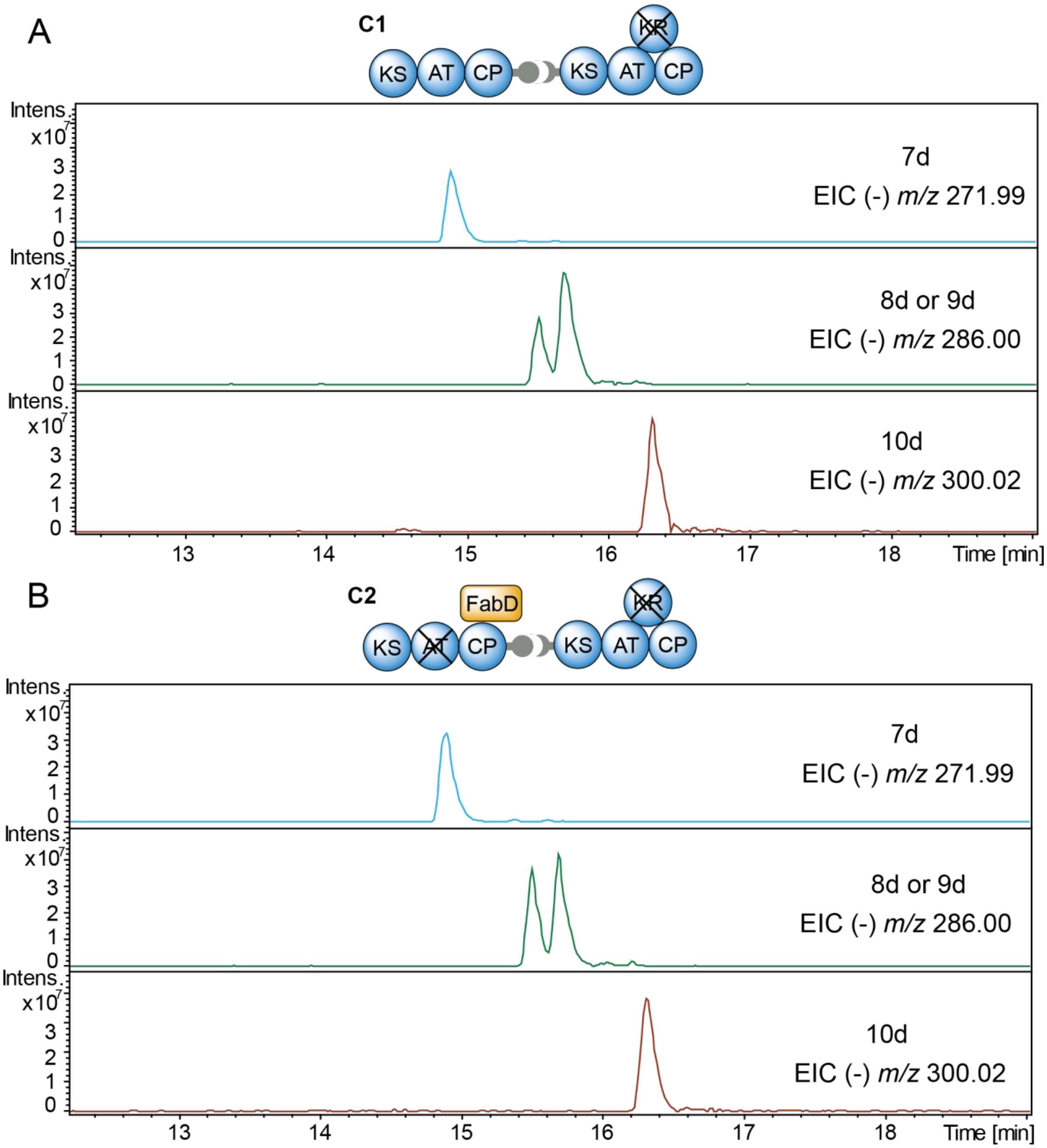

Figure S19: LC/MS-based detection of molecules **7d–10d** in C1 (**A**) and C2 (**B**) competition assays. Molecule **7d** is identified by EIC corresponding to *m/z* 271.99 Da, **8d** and **9d** are identified by EIC corresponding to *m/z* 286.00 Da, and **10d** is identified by EIC corresponding to *m/z* 300.02 Da. Differentiating between **8d** and **9d** was enabled by their MS^2 fragmentation pattern as shown in Figure S20.

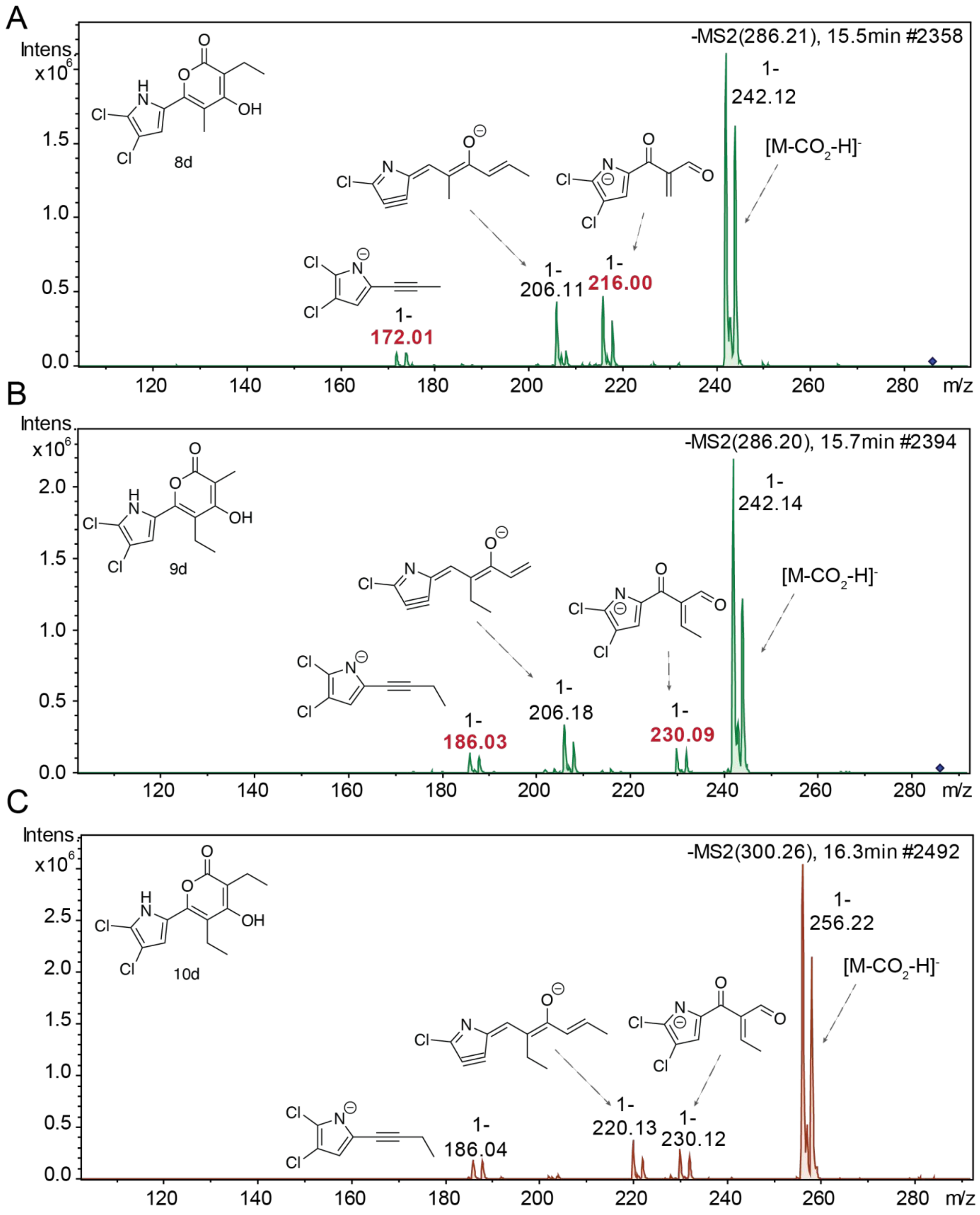

Figure S20: MS^2 spectra of propargyl pyrone **8d** (**A**), **9d** (**B**) and **10d** (**C**) with structural annotations of fragment ions. Ions highlighted in red represented fragment ions that only exist in one isomer, but not in the other one, which facilitated the annotation of isomer EIC peaks in Figure S19.

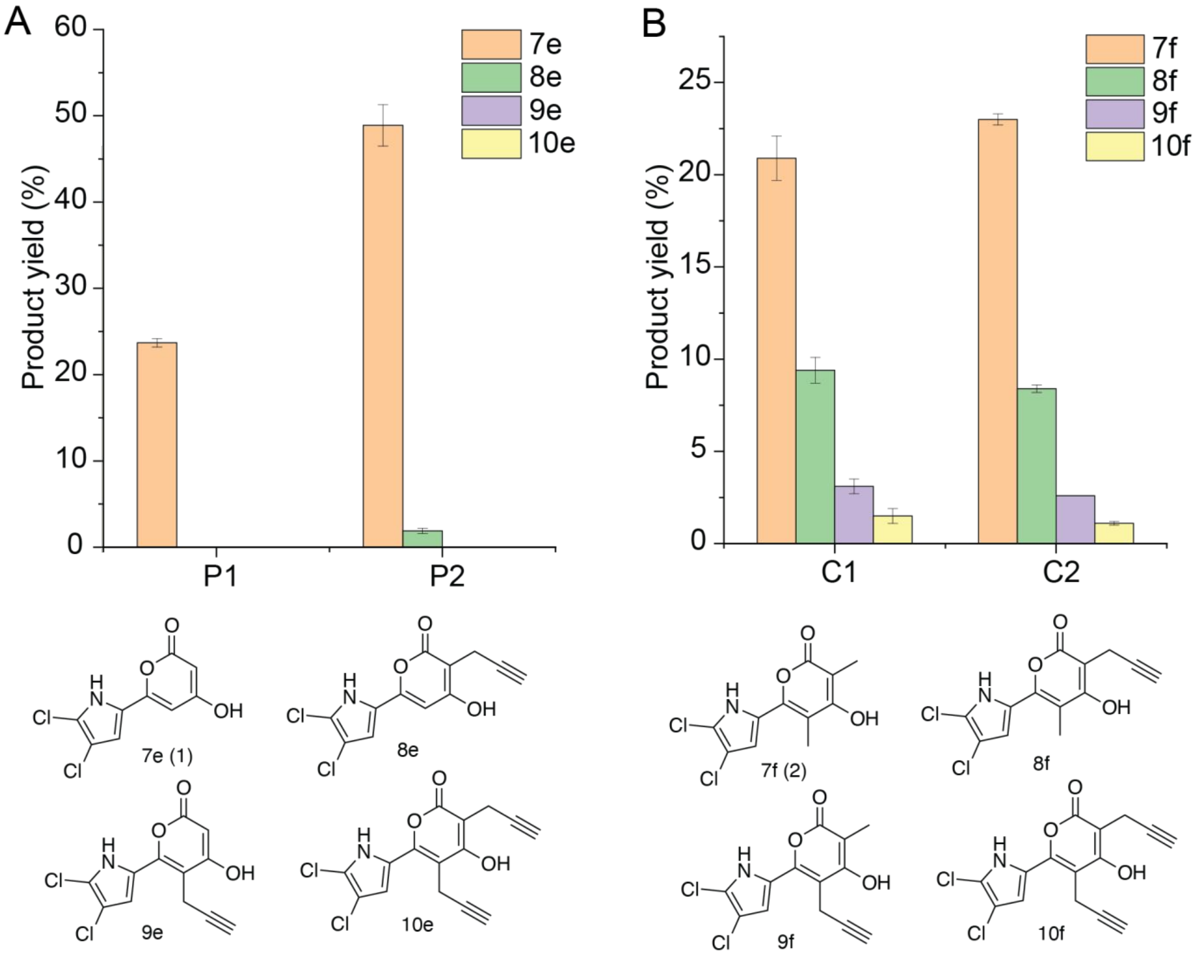

Figure S21: Product yield in the competition assay when PgMal-CoA was supplied as the non-native extender unit. (**A**) Production of pyrones **7e–10e** by P1 and P2 PKSs. Here, **7e** and **8e** comprise of 'Group A' products, and **9e** and **10e** comprise the 'Group B' products. (**B**) Production of pyrones **7f–10f** by C1 and C2 PKSs. Here, **7f** and **8f** comprise of 'Group A' products, and **9f** and **10f** comprise the 'Group B' products.

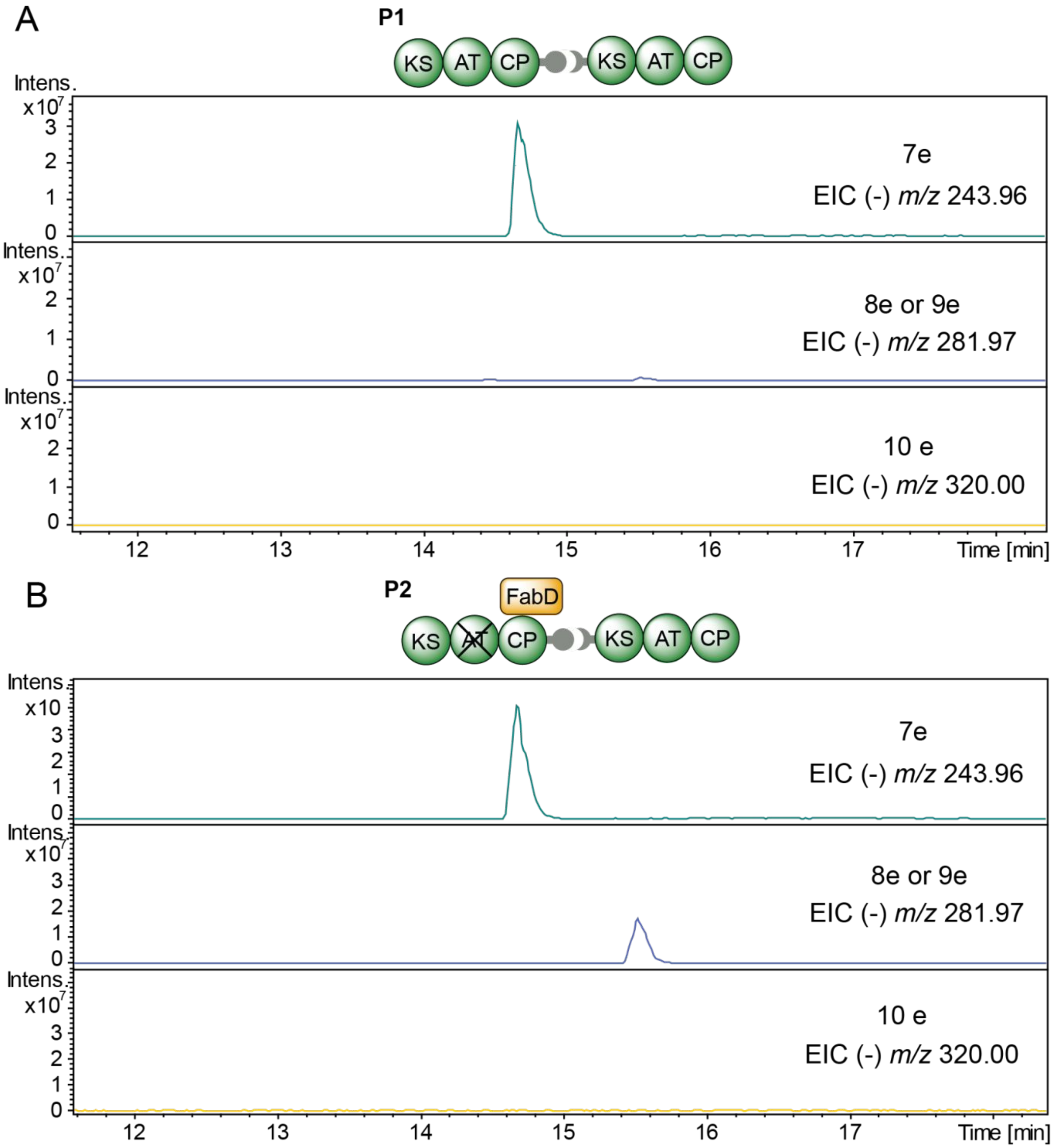

Figure S22: LC/MS-based detection of molecules **7e–10e** in P1 (**A**) and P2 (**B**) competition assays. Molecule **7e** is identified by EIC corresponding to *m/z* 243.96 Da, **8e** and/or **9e** are identified by EIC corresponding to *m/z* 281.97 Da, and **10e** is identified by EIC corresponding to *m/z* 320.00 Da. Differentiating between **8e** and **9e** was enabled by their MS^2 fragmentation pattern as shown in Figure S23.

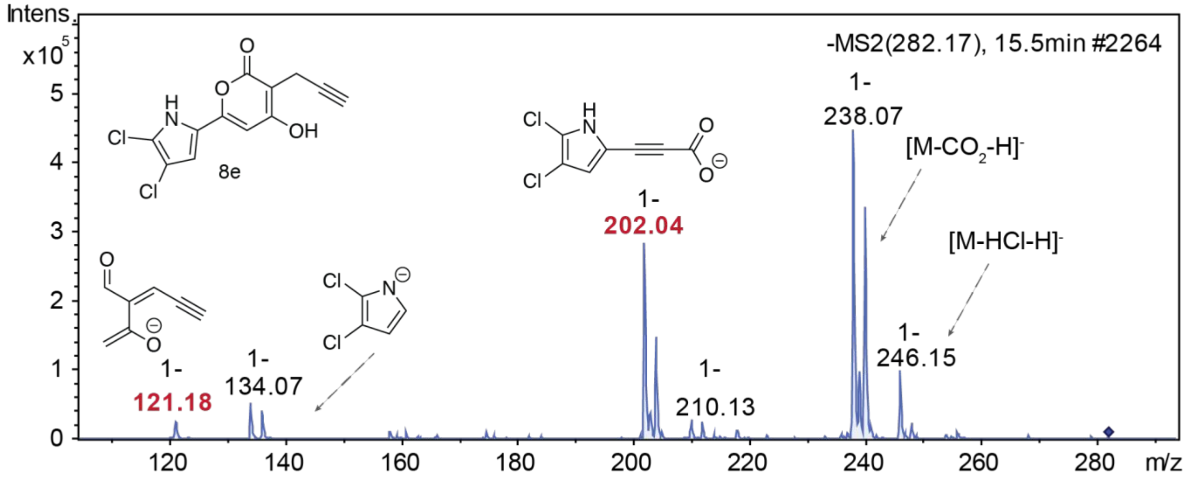

Figure S23: MS^2 spectra of **8e** with rationalized structural annotations of fragment ions. The presence of fragment ions highlighted in red demonstrated that the incorporation of the propargyl group occurred during the second elongation even to produce **8e**.

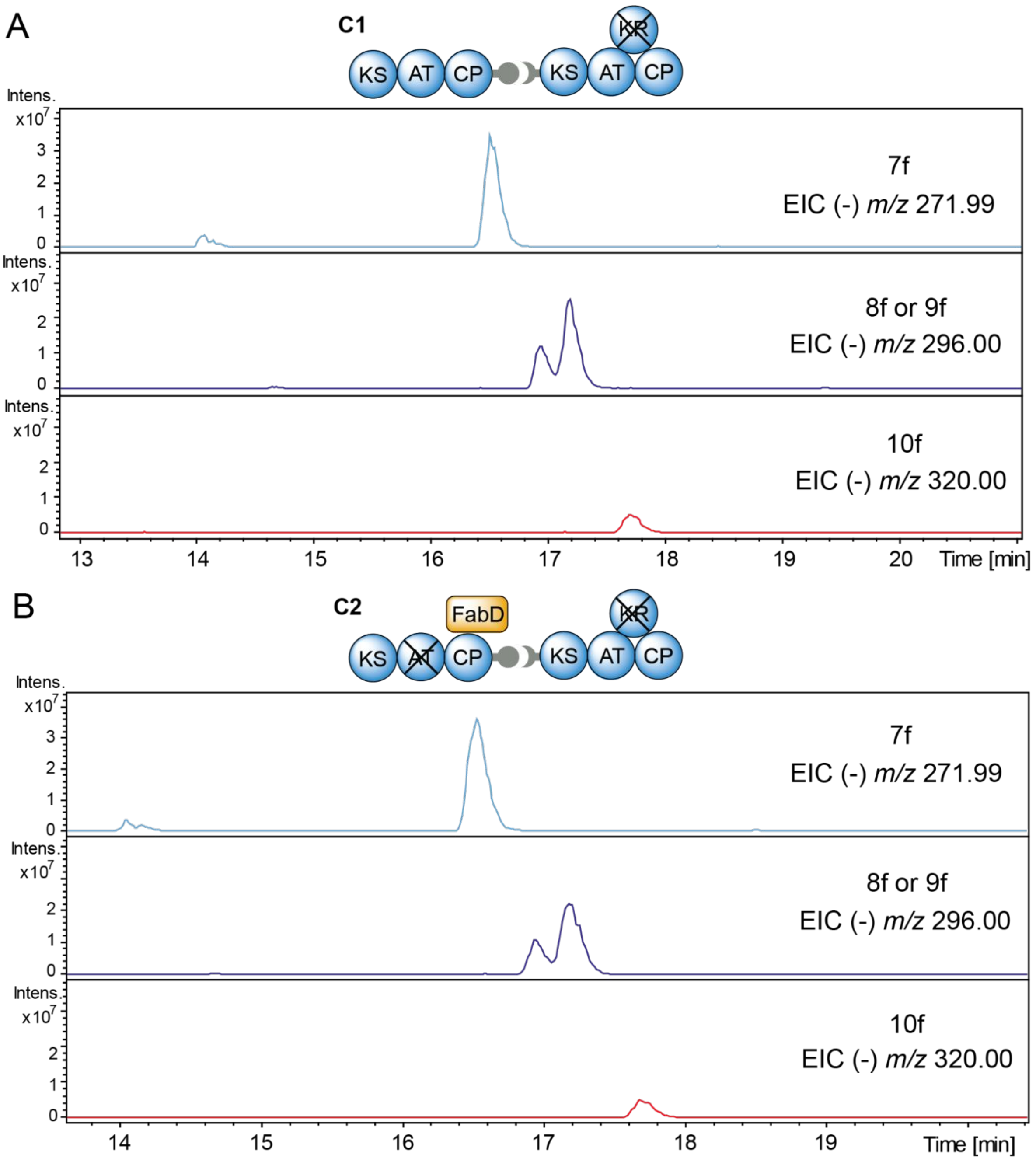

Figure S24: LC/MS-based detection of molecules **7f–10f** in C1 (**A**) and C2 (**B**) competition assays. Molecule **7f** is identified by EIC corresponding to *m/z* 271.99 Da, **8f** and **9f** are identified by EIC corresponding to *m/z* 296.00 Da, and **10f** is identified by EIC corresponding to *m/z* 320.00 Da. Differentiating between **8f** and **9f** was enabled by their MS^2 fragmentation pattern as shown in Figure S25.

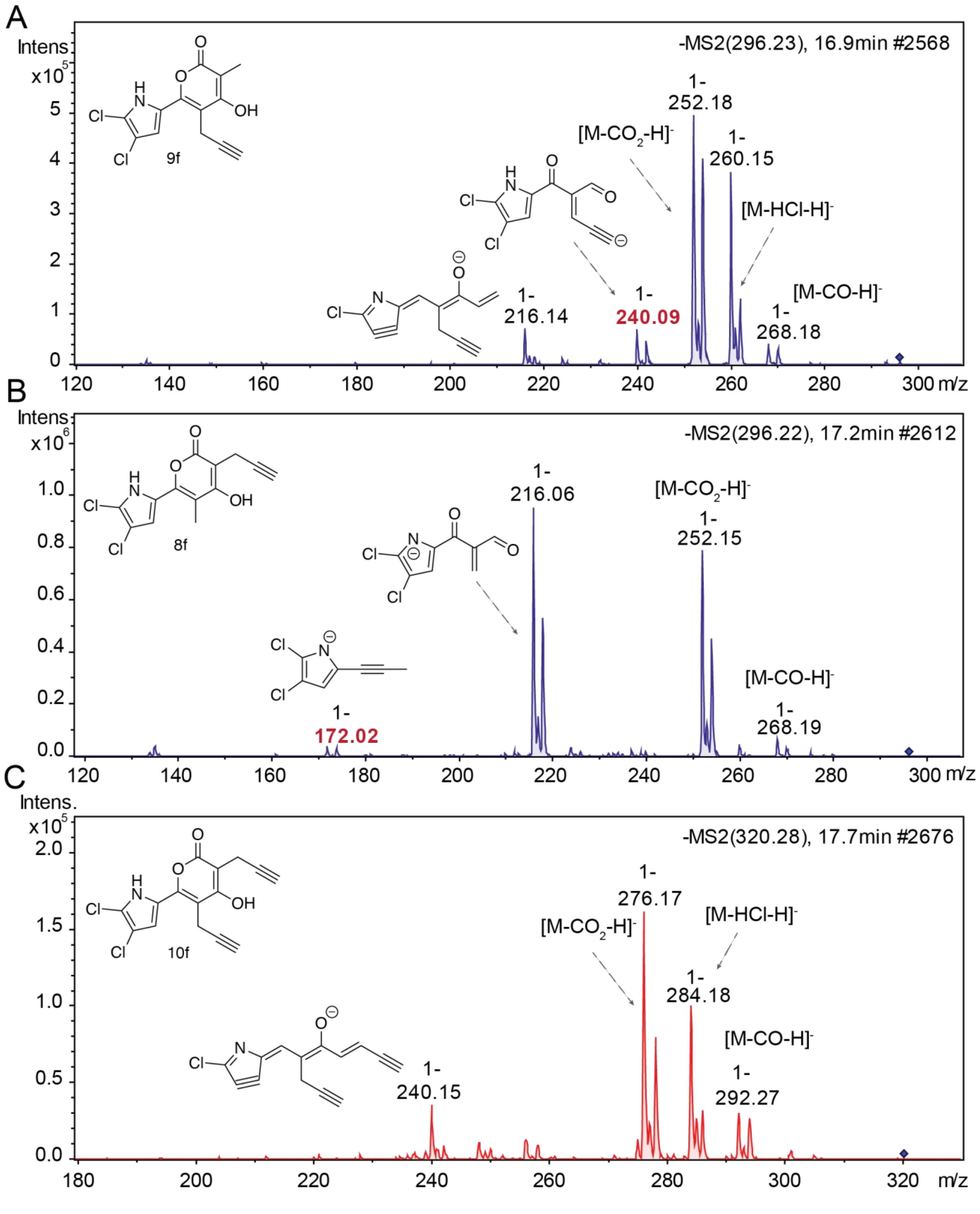

Figure S25: MS^2 spectra of **8f** (**A**), **9f** (**B**) and **10f** (**C**) with rationalized structural annotations of fragment ions. Ions highlighted in red represented fragment ions that only exist in one isomer, but not in the other one, which facilitated the annotation of isomer EIC peaks in Figure S24.

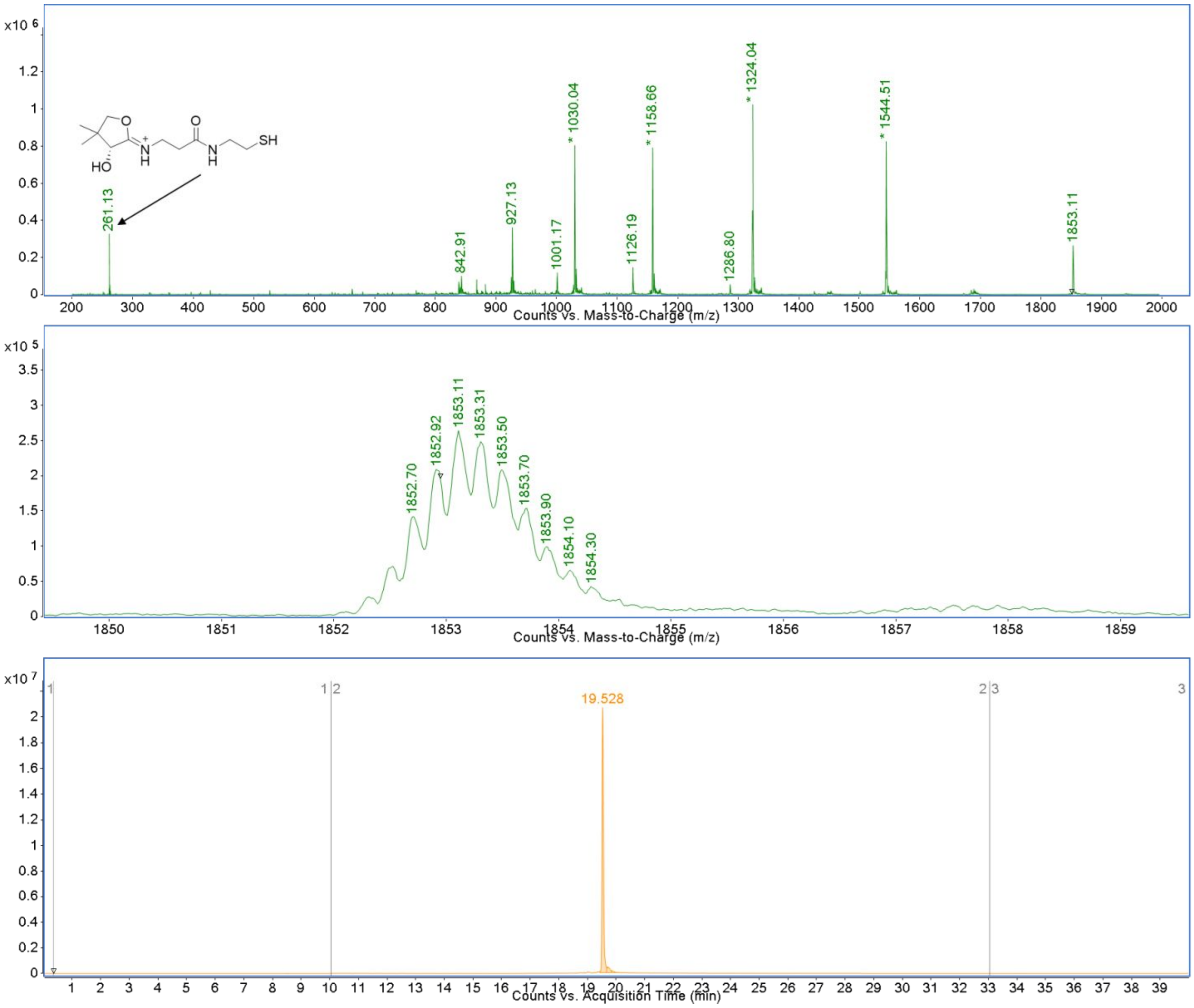

Figure S26: Phosphopantetheine ejection ion assay to characterize *E. coli* holo-CP. The *E. coli* apo-CP was recombinantly expressed and purified as a N-His$_6$ fusion protein. The apo-CP was converted to holo-CP *in vitro* using Sfp as described previously.[1] The thusly prepared holo-CP was digested with trypsin and analyzed by LC/MS using the phosphopantetheine ejection assay.[4-6] In this experiment, all MS^1 ions are subjected to an uniform fragmentation collision energy of 10 eV which generates a pseudo MS^2 spectra wherein the phosphopantetheine ejection ions are detected concomitantly with the peptide MS^1 ions. (**top**) MS^1 spectra for trypsin digested holo-CP. Note that the ion observed at *m/z* 261.13 represents the phosphopantetheine ejection ion as depicted. Other ions (*m/z* 842.91, 27.13, 1030.04, 1158.66, 1324.04, 1544.51, and 1853.11) represent different charge states for holo-CP, see below) (**middle**) Zoomed in view of the isotopic signature of the *m/z* 1853.11 ion which denotes the holo-CP in the $z = +5$ charge state. From here, accounting for the mass increase upon post-translational phosphopantetheinylation, the trypsin digested sequence of the CP can be deconvoluted to be:

GSHMSTIEERVKKIIGEQLGVKQEEVTNNASFVEDLGADSLDTVELVMALEEEFDTEIPDEEAEKI TTVQAAIDYINGHQA wherein the underlined Ser residue should be site for modification. Note that the ejection ion and the peptide ion are detected in the same spectra because the ejection ion is being generated by in-source fragmentation. Hence, data-dependent MS^2 spectra acquisition is not needed and MS^1 data acquisition can proceed at a high scan rate which is essential for high-fidelity relative quantification of different acyl-CP species. (**bottom**) MS^1 EIC demonstrating the chromatographic retention of the holo-CP. Note that the area under the EIC peak can be used for quantification of the relative abundances of different acylated states of the *E. coli* CP.

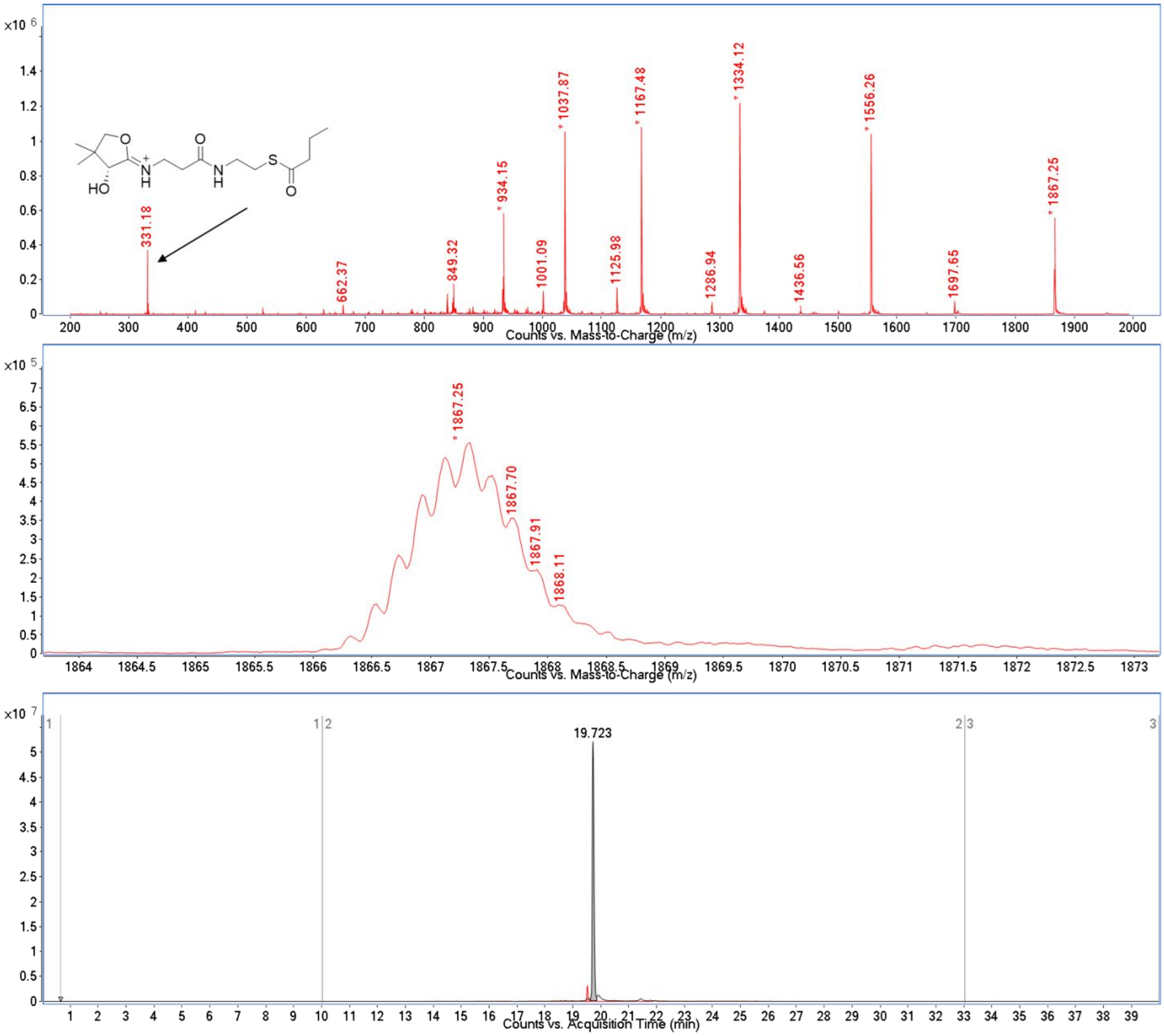

Figure S27: Phosphopantetheine ejection ion assay to characterize *E. coli* EtMal-CP synthesis using FabD. In this experiment, the ethylmalonyl (EtMal) moiety from MatB-generated EtMal-CoA was transferred to holo-CP *in vitro* using FabD. The extent of the FabD-catalyzed reaction is being monitored by determining the relative abundances of holo-CP (substrate) and EtMal-CP (product). (**top**) MS^1 spectra for trypsin digested EtMal-CP. Note that the ion observed at *m/z* 331.18 represents the decarboxylated form of EtMal-phosphopantetheine ejection ion as depicted. Decarboxylation is expected to proceed under the in-source fragmentation conditions of the LC/MS experiment. (**middle**) Zoomed in view of the isotopic signature of the *m/z* 1867.25 ion which denotes the decarboxylated EtMal-CP in the $z = +5$ charge state. (**bottom**) MS^1 EICs demonstrating the chromatographic retention of the holo-CP (in red) and the decarboxylated EtMal-CP (in black). Areas under the two EICs allow for relative quantification of the two acyl-CP species. In this

experiment, the EtMal-CP abundance was thusly determined to be 96.1%. Note that only the decarboxylated form of EtMal is detected here for the MS^1 peptide ion as well as for the ejection ion.

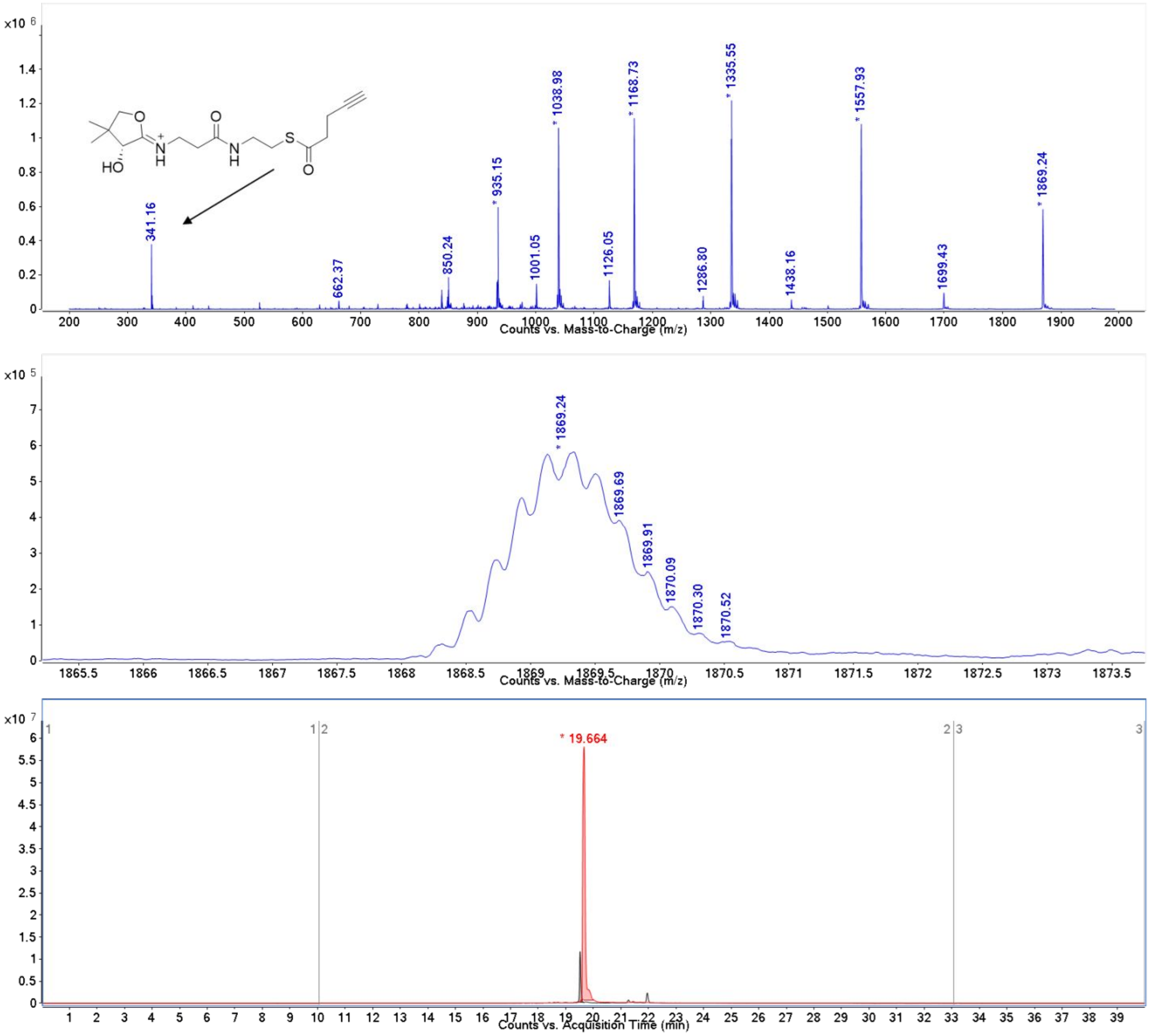

Figure S28: Phosphopantetheine ejection ion assay to characterize *E. coli* PgMal-CP synthesis using FabD. In this experiment, the proparagylmalonyl (PgMal) moiety from MatB-generated PgMal-CoA was transferred to holo-CP *in vitro* using FabD. The extent of the FabD-catalyzed reaction is being monitored by determining the relative abundances of holo-CP (substrate) and PgMal-CP (product). (**top**) MS^1 spectra for trypsin digested PgMal-CP. Note that the ion observed at *m/z* 341.16 represents the decarboxylated form of PgMal-phosphopantetheine ejection ion as depicted. Decarboxylation is expected to proceed under the in-source fragmentation conditions of the LC/MS experiment. (**middle**) Zoomed in view of the isotopic signature of the *m/z* 1869.24 ion which denotes the decarboxylated PgMal-CP in the z = +5 charge state. (**bottom**) MS^1 EICs demonstrating the chromatographic retention of the holo-CP (in black) and the decarboxylated PgMal-CP (in red). Areas under the two EICs allow for relative quantification of the two acyl-CP species. In this experiment, the PgMal-CP abundance was thusly determined to be 90.0%. Note

that only the decarboxylated form of PgMal is detected here for the MS^1 peptide ion as well as for the ejection ion.

Supplementary References

1. Yi, D.; Agarwal, V., Biosynthesis-guided discovery and engineering of α-pyrone natural products from type I polyketide synthases. *ACS Chemical Biology* **2023,** *18* (5), 1060-1065.

2. Hughes, A. J.; Keatinge-Clay, A., Enzymatic extender unit generation for in vitro polyketide synthase reactions: structural and functional showcasing of *Streptomyces coelicolor* MatB. *Chem Biol* **2011,** *18* (2), 165-76.

3. Forte, N.; Benni, I.; Karu, K.; Chudasama, V.; Baker, J. R., Cysteine-to-lysine transfer antibody fragment conjugation. *Chemical Science* **2019,** *10* (47), 10919-10924.

4. Agarwal, V.; Diethelm, S.; Ray, L.; Garg, N.; Awakawa, T.; Dorrestein, P. C.; Moore, B. S., Chemoenzymatic synthesis of acyl coenzyme A substrates enables *in situ* labeling of small molecules and proteins. *Org Lett* **2015,** *17* (18), 4452-5.

5. Dorrestein, P. C.; Bumpus, S. B.; Calderone, C. T.; Garneau-Tsodikova, S.; Aron, Z. D.; Straight, P. D.; Kolter, R.; Walsh, C. T.; Kelleher, N. L., Facile detection of acyl and peptidyl intermediates on thiotemplate carrier domains via phosphopantetheinyl elimination reactions during tandem mass spectrometry. *Biochemistry* **2006,** *45* (42), 12756-66.

6. Dorrestein, P. C.; Kelleher, N. L., Dissecting non-ribosomal and polyketide biosynthetic machineries using electrospray ionization Fourier-Transform mass spectrometry. *Nat Prod Rep* **2006,** *23* (6), 893-918.
